# Methods to Develop an *in silico* Clinical Trial: Computational Head-to-Head Comparison of Lisdexamfetamine and Methylphenidate

**DOI:** 10.3389/fpsyt.2021.741170

**Published:** 2021-11-03

**Authors:** José Ramón Gutiérrez-Casares, Javier Quintero, Guillem Jorba, Valentin Junet, Vicente Martínez, Tamara Pozo-Rubio, Baldomero Oliva, Xavier Daura, José Manuel Mas, Carmen Montoto

**Affiliations:** ^1^Unidad Ambulatoria de Psiquiatría y Salud Mental de la Infancia, Niñez y Adolescencia, Hospital Perpetuo Socorro, Badajoz, Spain; ^2^Servicio de Psiquiatría, Hospital Universitario Infanta Leonor, Universidad Complutense, Madrid, Spain; ^3^Anaxomics Biotech, Barcelona, Spain; ^4^Research Programme on Biomedical Informatics (GRIB), Departament de Ciències Experimentals i de la Salut, Universitat Pompeu Fabra, Barcelona, Spain; ^5^Institute of Biotechnology and Biomedicine, Universitat Autònoma de Barcelona, Cerdanyola del Vallès, Spain; ^6^Medical Department, Takeda Farmacéutica España, Madrid, Spain; ^7^Catalan Institution for Research and Advanced Studies (ICREA), Barcelona, Spain

**Keywords:** attention-deficit/hyperactivity disorder, lisdexamfetamine, methylphenidate, mathematical modeling, *in silico* clinical trial

## Abstract

Regulatory agencies encourage computer modeling and simulation to reduce the time and cost of clinical trials. Although still not classified in formal guidelines, system biology-based models represent a powerful tool for generating hypotheses with great molecular detail. Herein, we have applied a mechanistic head-to-head *in silico* clinical trial (ISCT) between two treatments for attention-deficit/hyperactivity disorder, to wit lisdexamfetamine (LDX) and methylphenidate (MPH). The ISCT was generated through three phases comprising (i) the molecular characterization of drugs and pathologies, (ii) the generation of adult and children virtual populations (vPOPs) totaling 2,600 individuals and the creation of physiologically based pharmacokinetic (PBPK) and quantitative systems pharmacology (QSP) models, and (iii) data analysis with artificial intelligence methods. The characteristics of our vPOPs were in close agreement with real reference populations extracted from clinical trials, as did our PBPK models with *in vivo* parameters. The mechanisms of action of LDX and MPH were obtained from QSP models combining PBPK modeling of dosing schemes and systems biology-based modeling technology, i.e., therapeutic performance mapping system. The step-by-step process described here to undertake a head-to-head ISCT would allow obtaining mechanistic conclusions that could be extrapolated or used for predictions to a certain extent at the clinical level. Altogether, these computational techniques are proven an excellent tool for hypothesis-generation and would help reach a personalized medicine.

## Introduction

To reduce clinical trials time and cost and to improve their outcomes' conclusiveness, regulatory agencies encourage the use of computer modeling and simulation (CM&S) approaches to optimize randomized clinical trials (1). CM&S approaches are based on the analysis of existing data and experience, including real-world data studies, pharmacometrics modeling or, more recently, *in silico* clinical trials (ISCT). Although the concept emerged in the early 2000s (2–4), the term and proper definition of ISCT was widely established and accepted during the 2010 decade with the foundation of specific organizations to promote the implementation of these approaches, such as the VPH Institute in 2011 or the Avicenna Alliance, founded by the European Commission, to create the research roadmap for ISCT (5). In addition to its economic advantages, ISCT allow the exploration of drugs and diseases in many settings, thus, reducing risks for patients and the use of animal models to test hypotheses. CM&S and artificial intelligence-based approaches are crucial to achieving personalized, preventive, predictive, participative, and precise—the so-called 5P—medicine and healthcare (6).

### Systems Biology and MID3 Guidelines

One of the most promising computational tools encompassing these concepts is systems biology or systems medicine (5, 7–9). During the last 20 years, the US and European medicines agencies (FDA and EMA), in collaboration with the pharmaceutical industry, have been developing the guidelines and good practices to which these computational approaches should adhere. One of these guidelines is MID3, which describes the quantitative framework for predicting and extrapolating models' conclusions (10, 11). Establishing three categories based on the relevance of the conclusions, MID3 is meant to guide industry decision-making (12) or regulatory assessment (13). Accordingly, models can be classified as (i) “LOW” impact, when information obtained from them cannot be directly used to make clinical or commercial decisions [e.g., physiologically based pharmacokinetic (PBPK)] models; (ii) “MEDIUM” impact, for models providing helpful information for strategic conditioning of future trial data [e.g., studies to determine optimal dosing, target population, sample size, design of future trials, or study of mechanisms of action (MoA) of compounds]; and (iii) “HIGH” impact, for cases where conclusions support decision-making without the need for additional experimental or trial studies (e.g., simulations replacing direct clinical trial data in children or oncologic patients that provide evidence on efficacy and safety to uphold regulatory submission package and labeling). While pharmacometric models are under evaluation for acceptance as HIGH impact models, systems biology-based models are still in debate (14). However, they possess an undeniable great potential in providing molecular detail, generating hypotheses, and suggesting specific molecular solutions to complex pathophysiological problems.

### Proof-of-Concept: ADHD

Attention-deficit/hyperactivity disorder (ADHD) is a complex ailment with a prevalence in children ranging from 6 to 10% (15). Besides, ADHD exhibits an important long-term persistence (15), affecting ~5% of adults (16–18). Around 30–50% of children with ADHD continue to manifest symptoms, inattention in particular, in adulthood (19, 20). Comorbid psychiatric disorders are present in up to 67% of ADHD pediatric-adolescent patients (21) and almost 80% of adults (22). These comorbidities can complicate ADHD diagnosis and treatment (23, 24) and include depression, anxiety, bipolar disorder, binge eating, tics, conduct disorder, personality disorder and non-alcoholic substance abuse, among others (20, 25). Recent findings suggest a direct relationship between ADHD and the development of these comorbidities (24, 26, 27), likely involving a genetic connection (28), although results on this subject remain controversial.

ADHD management comprises pharmacologic and non-pharmacologic treatments. Medications include stimulant [amphetamines and methylphenidate (MPH)] and non-stimulant drugs (atomoxetine, extended-release clonidine, and guanfacine), with the former being recommended as first-line treatment (29). Several modifications to improve the characteristics of amphetamines have been performed, among which the design of the prodrug lisdexamfetamine (LDX, Vyvanse^®^ in the US and Elvanse ^®^ in Europe) and the development of extended-release formulations [such as the osmotic release oral system (OROS) of MPH, Concerta^®^ or Medikinet^®^ retard]. Although a pediatric clinical trial analyzing LDX and MPH is currently ongoing (30, 31), there are no explicitly designed head-to-head trials comparing these treatments, neither on the pediatric nor adult population.

We present here the methods of the Therapeutic Performance Mapping System (TPMS) technology, which allow the generation of virtual patients and PBPK and systems biology-based models with the purpose of performing ISCTs. To demonstrate the applicability of the method, we used as case-study a mechanistic head-to-head ISCT between LDX and MPH (Elvanse^®^ vs. Concerta^®^ in the pediatric-adolescent population and Elvanse^®^ vs. Medikinet^®^ retard in the adult population) using a crossover–like design. The objective of this ISCT was to model the efficacies of the two drugs and compare them in a virtual head-to-head setting. Additionally, we describe an approach to measure and compare the output results in terms of efficacy of the two medications, the molecular mechanisms triggered, and the response to ADHD management in a diverse population of virtual patients, including patients with the most common psychiatric comorbidities.

## Methods

**Figure 1:**
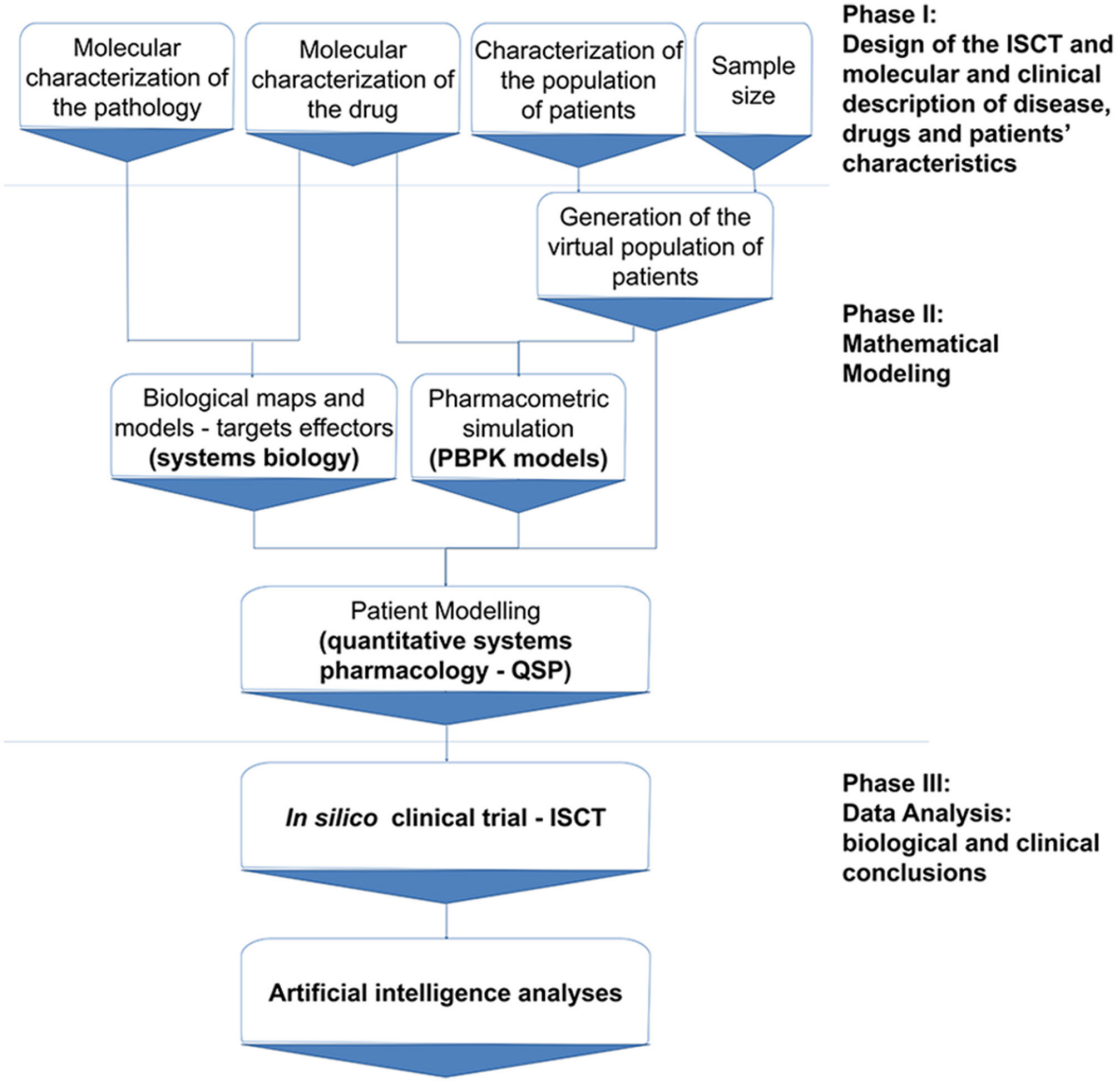
*In silico* clinical trial protocol overview. The protocol is divided into three main stages: Phase I, including trial design and information compilation; Phase II, comprising mathematical modeling; and Phase III, consisting of data analysis according to the trial design. ISCT, *in silico* clinical trial; PBPK, Physiologically based pharmacokinetic; QSP, Quantitative systems biology.

This methods study details the steps and modeling approaches to carry out the ISCT (Figure 1). Before the study trial (phase I), drugs and pathological conditions were molecularly characterized and reference populations defined. In the modeling stage (phase II), a series of virtual populations and PBPK and quantitative systems pharmacology (QSP) models were generated and embedded in the ISCT as a means of virtual patient recruitment. At this step, the models were optimized to reproduce known clinical efficacy findings according to the primary outcome of the study, i.e., the model-based clinical efficacy-related measure herein proposed, based on modeled protein activity over ADHD molecular definition. Finally, in the analysis phase (phase III), the molecular variability among patients was explored by analyzing all ADHD models, patient by patient.

### Population Definition—Virtual Patients

Two types of virtual populations (vPOPs) were generated: adult (>18 years old) and pediatric-adolescent (6–17 years old) vPOPs. As reference demographic and comorbidity parameters to generate the VPOPs, the following studies were used: NCT00730249 (32) (MPH) and NCT00337285 (33) (LDX) for adults; and NCT00763971 study (30) (LDX and MPH) for the pediatric-adolescent population. These clinical trials presented standard inclusion and exclusion criteria for ADHD evaluation, which were appropriate for the case-study herein proposed and showed homogeneous demographic values when compared to other clinical trials with equivalent inclusion and exclusion criteria.

Additionally, standard population distribution data was used to fill incomplete demographic parameters. For adults, ESS Round 7 (34) was used, while data from the World Health Organization (WHO) growth information (35) was retrieved for the pediatric-adolescent population.

All virtual patients created had ADHD, and specific branches for the different comorbidities were also generated, as previously described (36). ADHD and comorbidities definitions were obtained by thorough literature review of current molecular knowledge on each condition (see Supplementary Methods in Supplementary Material 1; Supplementary Tables A, B in Supplementary Material 2).

#### Sample Size Calculation

Since data on treated and non-treated patients is not available, we considered that a number of patients large enough to discriminate among ADHD patients and healthy individuals would also be large enough to detect efficacy-associated changes for each drug. Therefore, to generate enough patients and ensure having sufficient statistical power when performing data analyses, the sample size approach described below was carried out. Because TPMS' drug efficacy outcomes are based on predicted protein activity (i.e., tSignal, Equation 1—defined in section Systems Biology Maps and Models), this methodology was based on experimental measures that can relate to protein activity variability, particularly gene expression.

First, gene expression data groups identified as control- “healthy” and case- “disease” were retrieved from Gene Expression Omnibus (GEO) experiments (37) and then treated and normalized using R packages, parameters, and steps defined by Law et al. (38). Afterwards, a protocol based on the method introduced by Mukherjee et al. (39) and Figueroa et al. (40) was followed to explore the variation in accuracy and statistical power induced by changes in the sample size. To that end, GEO patient-normalized gene expression datasets are submitted to sampling-without-replacement combined with a linear regression classification method (41). The latter allows the identification of the best classifiers (proteins) to separate control-healthy from case-disease patients, and these classifiers are used to compute the highest possible accuracy (“Max accuracy”). Progressive sampling is then applied to obtain subsets of balanced samples from both cohorts (case-disease vs. control) in a 1:1 ratio. These subsets are tested for sample sizes ranging from eight to the number of the smallest cohort performing 100 repetitions per sample size. Each subset is used to train a linear classifier based on two features extracted by feature selection procedures previously described (36). The accuracy achieved for each classifier is estimated using *k*-fold cross-validation (*k* = 10) (42). Finally, taking as reference the Max accuracy, the percentage of max accuracy reached for each subset of samples and total samples is calculated using the classifiers obtained for that subset.

**Figure 2:**
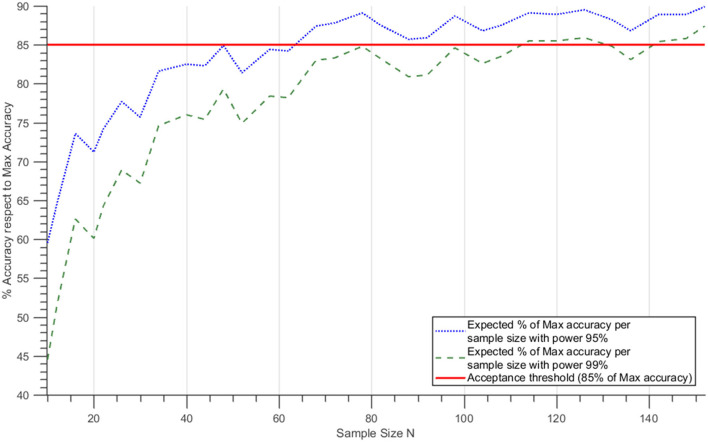
Expected percentage of best accuracy as a function of sample size. Dotted blue and discontinuous green lines correspond to the mean % best accuracy reached for each sample size at statistical power 95 and 99%, respectively, assuming a normal distribution of the accuracy variation and estimating the means and the standard deviation for each sample size. The red line shows the 85% Max accuracy level.

For the present ADHD study-case, RNAseq records from the entry GSE159104 (43) were selected, where two cohorts of patients were already identified and labeled as control (healthy) and ADHD (case-disease). The variability within the genes or proteins involved in the ADHD molecular definition (see Supplementary Methods in Supplementary Material 1; Supplementary Tables A, B in Supplementary Material 2) was evaluated for the 154 samples (78 control, 76 ADHD) included in the GEO experiment. After finalizing the abovementioned procedure, statistical powers of 95 and 99% were used, based on classification errors (39), and a value of 85% of Max accuracy was set as minimum valid threshold (Figure 2). Considering a statistical power of 95%, we deemed 68 samples (34 control and 34 ADHD) to be enough to achieve the objectives of the analysis in our simulation. Under these premises, 142 samples (71 control and 71 ADHD) were adequate to reach the target accuracy with 99% power (although more RNAseq samples would be required to ensure curve stabilization). Accordingly, at least 100 virtual patients were built per each patient group (minimum sample size of 200 samples per analysis).

#### Patient Distribution

The two populations, adult and pediatric-adolescent, were segmented into nine arms each (a total of 18 arms) to facilitate the simulation and the analysis. One arm accounted for ADHD without any comorbidity, while the eight additional arms contained patients with ADHD and one, or a combination, of comorbid psychiatric conditions.

**Figure 3:**
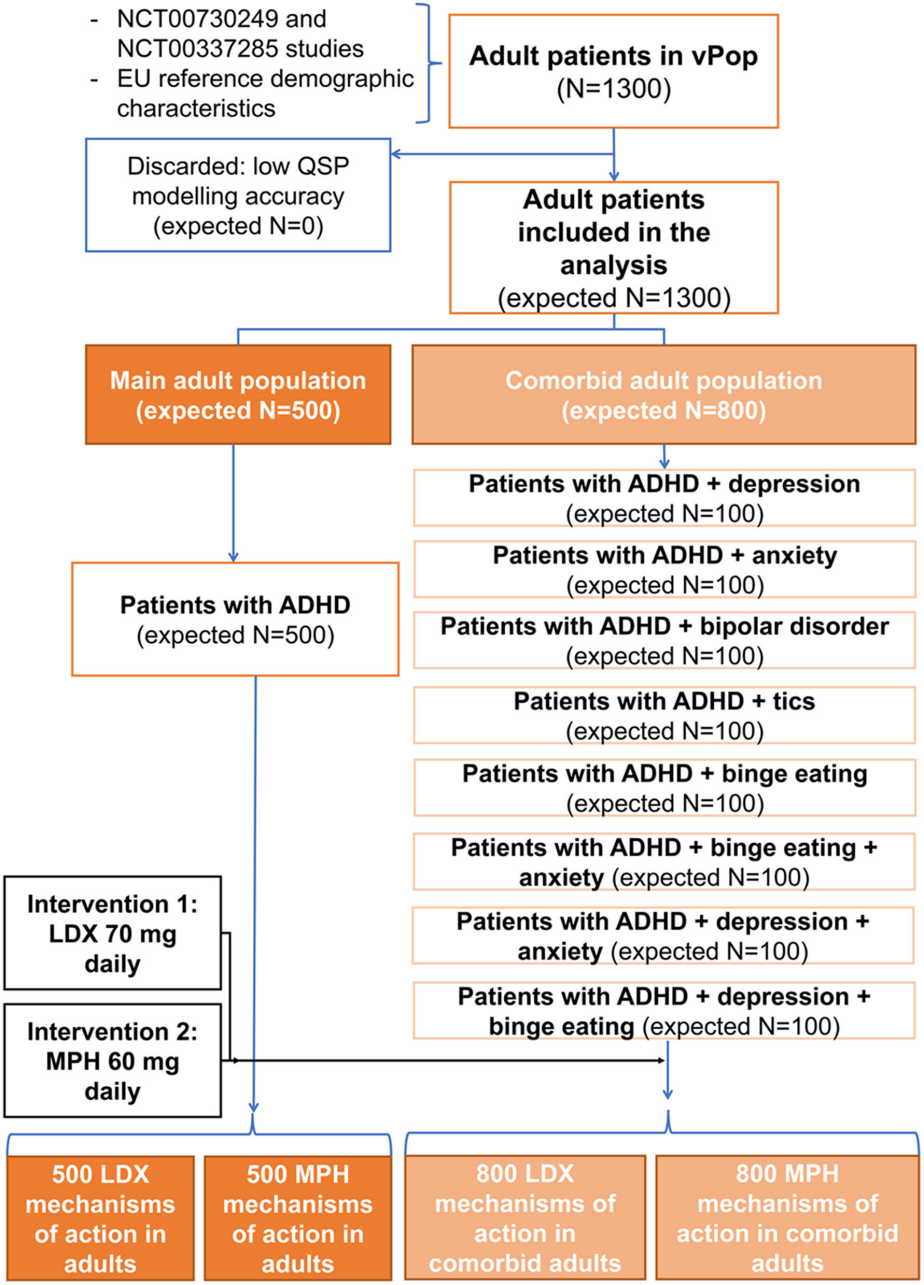
Comorbidities distribution and treatment allocation in the adult virtual population. ADHD, Attention-deficit/hyperactivity disorder; LDX, Lisdexamfetamine; MPH, Methylphenidate; QSP, Quantitative systems biology; vPOP, Virtual population.

**Figure 4:**
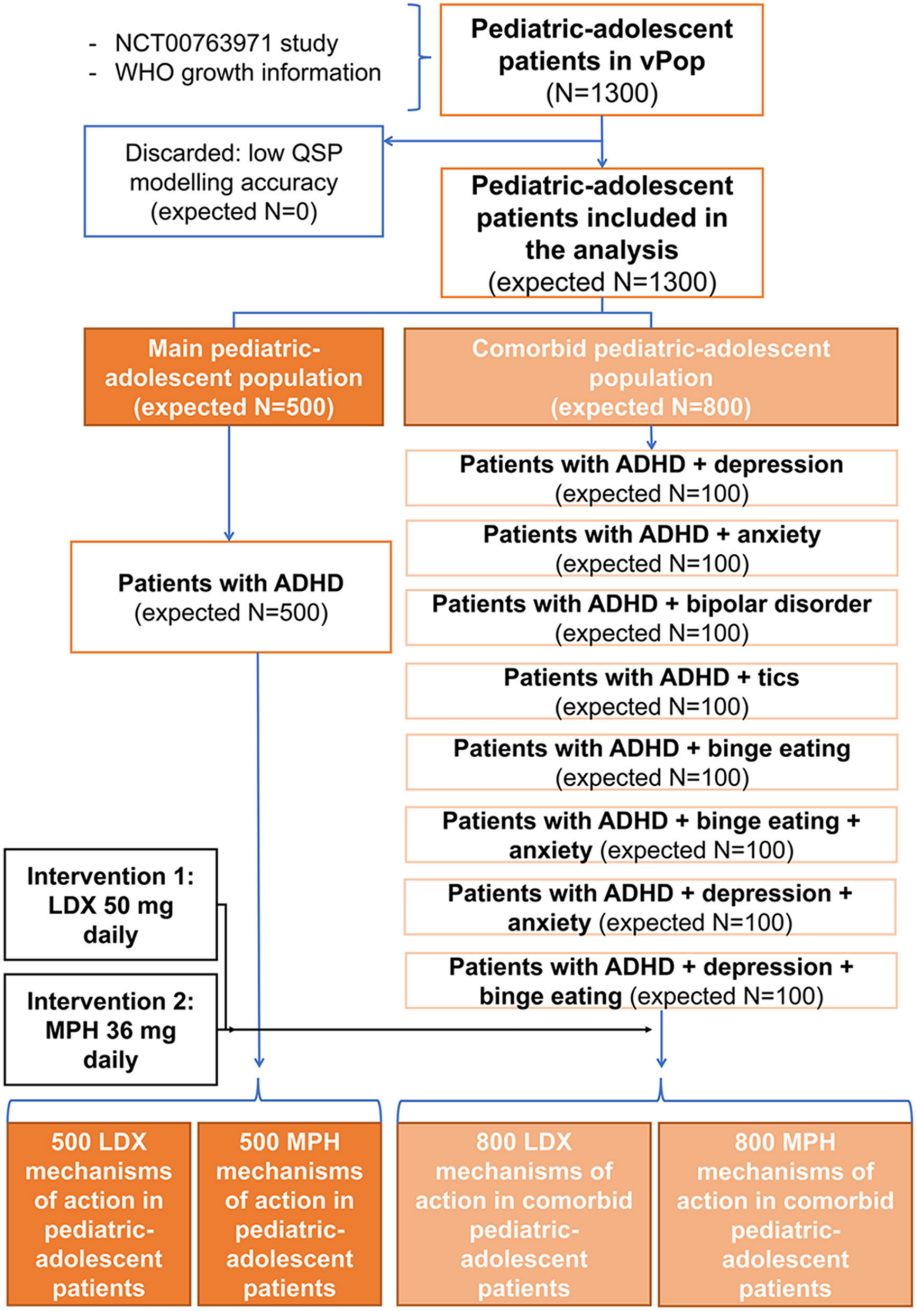
Comorbidities distribution and treatment allocation in the pediatric-adolescent virtual population. ADHD, Attention-deficit/hyperactivity disorder; LDX, Lisdexamfetamine; MPH, Methylphenidate; QSP, Quantitative systems biology; vPOP, Virtual population.

Each of the arms accounting for comorbidities had 100 patients, while arms related to ADHD alone consisted of 500 patients, with the aim of maximizing the number of patients with different demographical characteristics. Consequently, a total of 2,600 patients were included in the simulation: 1,300 adults (Figure 3) and 1,300 children-adolescents (Figure 4). All of them were treated sequentially with LDX and MPH using the adequate dosing scheme. According to the study's *in silico* nature, the files containing the models of each virtual patient could be cloned; thus, no wash-out period was needed.

### Intervention Definition

According to their population group, the patients included in the ISCT were treated with different formulations and doses of LDX and MPH in a two-period crossover-like study design. For all patients, the same initial state was used at each period, hence carryover effect was assumed zero. Dosage, molecular target profile, and pharmacokinetic information were needed for the QSP modeling herein proposed.

#### Dosage

Dosage schemes were simulated differently in the pediatric-adolescent and adult populations according to usual clinical practices. Adults were treated with LDX (Elvanse^®^) 70 mg and children with LDX 50 mg. Different doses and types of modified release systems for MPH were considered in the simulation, corresponding to different commercial formulations: (i) for adults, Medikinet^®^ 60 mg with modified-release (also known as Medikinet^®^ XL or Medikinet^®^ Retard), based in multiarticular beads that combine 50% immediate and 50% extended-release (44); and (ii) for the pediatric-adolescent population, Concerta^®^ 36 mg, an osmotic release system (OROS technology) with a 22% of the total amount available for immediate release (the remaining 78% corresponding to the osmotically controlled extended-release) (45).

#### Molecular Target Profile

The molecular target profile identification was performed through a review of official regulatory sources [European Medicines Agency—EMA, European Public Assessment Report (EPAR)—and Food and Drug Administration—FDA, Multidisciplinary and Chemistry reviews and Label], drug-target–dedicated databases [DrugBank (46), STITCH (47), SuperTarget (48)] and the scientific literature (the specific searches performed can be found in Supplementary Methods in Supplementary Material 1). This information was integrated into the TPMS technology-based MoA models for each drug. Table 1 contains the proteins defining the target profile of LDX and MPH.

**Table 1:** Identified protein targets for lisdexamfetamine and methylphenidate.

**Gene name**	**Protein name**	Effect^*^	**Reference of LDX target**	**Reference of MPH target**
TAAR1	Trace amine-associated receptor 1	1	(49)	–
SLC18A2	Synaptic vesicular amine transporter (VMAT2)	−1	(49, 50)	–
SLC6A3	Sodium-dependent dopamine transporter (DAT)	−1	(50–52)	(53–55)
SLC6A2	Sodium-dependent noradrenaline transporter (NET)	−1	(50, 52)	(53–55)
SLC6A4	Sodium-dependent serotonin transporter (SERT)	−1	(50)	–
MAOA	Amine oxidase (flavin-containing) A	−1	(52, 56)	–
MAOB	Amine oxidase (flavin-containing) B	−1	(52, 56)	–
HTR1A	5-hydroxytryptamine receptor 1A	1	–	(57, 58)

* *Effect refers to the drug's action on the protein, 1 denotes activation of protein function, −1 inhibition of protein function*.

*LDX, lisdexamfetamine; MPH, methylphenidate*.

#### Pharmacokinetics Information

Bioavailability and drug's information on main clearance organ were retrieved from published studies and set for the corresponding PBPK models (Table 2). Moreover, previous PK studies were used to fit the generated PBPK models, to parameterize absorption and drugs' clearance ratios, and to validate the models. The reference studies used were Krishnan and Zhang (66) for LDX in adults, Boellner et al. (67) for LDX in children, the EPAR (68) for Medikinet^®^ with modified-release, and Maldonado (69) for Concerta^®^. All three drugs were administered orally and crossed the blood-brain barrier.

**Table 2:** Summary of pharmacometrics information used for PBPK modeling.

**Drug**	**% Bioavailability (Ref.)**	**Main clearance organ**
Elvanse^®^	96.4 (59)	Kidney (60)
Medikinet^®^ with modified release	30 (61, 62)	Kidney (63)
Concerta^®^	32 (64)	Kidney (63, 65)

### Modeling Methodology

TPMS ISCT is divided into three types of modeling approaches (Figure 1). First, virtual patients are generated containing demographic information and disease tags. Afterwards, PBPK models are constructed using each patient's demographic variables, which are then used to infer inter-patient specific drug concentration-related knowledge. Finally, the patient-specific drug concentration and disease-related data, and protein mapping according to pathophysiological information, are used for generating patient-specific MoA-QSP models of the drugs under study, here MPH and LDX.

#### Virtual Population Modeling

For the construction or recruitment of vPOPs, randomized populational demographic characteristics are generated using two types of data sources: (i) original or reference population with demographic characteristics to be mimicked [age, weight, height, and/or body mass index (BMI)]; and (ii) standard population distributions, retrieved from populational studies. For the present ADHD study-case, the recruitment of each vPOP was based on the demographical parametric descriptors defined in section Population Definition—Virtual Patients' [reference clinical trials (30, 32, 33), European standard population (34), and WHO growth information (35)].

For adult population, an adapted version of the algorithm proposed by Allen et al. (70) was used to generate the population of individuals virtually recruited in the trial. As a first step, this algorithm generates a multivariate normal distribution (MVND) with the demographic means and standard deviations from the original population. The standard population distribution values are used to fill in the potential missing demographic information. A simulated annealing strategy is then used to minimize a cost function by using the patients generated in the MVND as starting points (see Supplementary Methods in Supplementary Material 1).

In the pediatric-adolescent population, a modification of the protocol used for adult population was applied to adjust better the dependence of morphometric measures for ages 0–17 years. First, the standard population distribution, taken from the growth information published by the WHO (35), was used to create a reference MVND. Then, a sampling strategy based on a Metropolis-Hastings method (71) was applied to reach the original population distributions (see Supplementary Methods in Supplementary Material 1).

The final distribution values for adult and pediatric-adolescent populations were statistically compared (one sample *z*-test) to the original means and standard deviations; only populations not significantly different from the original population (*p*-value > 0.05) were accepted and kept for posterior modeling steps. For both population types, corresponding comorbidity-related tags were assigned to the patients allocated to each of the 18 ISCT arms (Figure 4).

Demographic parameters were used to obtain accurate and individualized PBPK models of the drugs, while comorbidity data, once translated into molecular information, influenced the patients' corresponding QSP models.

#### Systems Biology Maps and Models

TPMS technology (36) generates mathematical models that use known biological, medical, and pharmacological information as training data (see Supplementary Table C in Supplementary Material 2) to simulate the behavior of drugs and the pathophysiology of diseases in terms of changes in protein activity. This methodology uses supervised machine learning methods based on a human protein functional network to infer information at the clinical and protein levels. Here, TPMS was used to build the mathematical models to simulate the behavior of LDX and MPH over ADHD by modeling the changes in proteins' activity defining the disease. While generating TPMS models, molecular information relating to psychiatric comorbidities was added to denote the different neurophysiological ADHD patient types.

The resulting models allowed the extraction of several protein activity measures. Therefore, the model-derived parameter tSignal (Equation 1) (36), which ranges between 1 and −1, applied to the molecular definition of clinical conditions (in this case, ADHD molecular definition, as detailed in Supplementary Table B in Supplementary Material 2) permitted access to clinically relevant information at a model-patient level.


(1) $$\begin{array}{r} tSignal=\;-\frac{1}{n}\sum_{i=1}^{n}v_{i}y_{i} \end{array}$$

Where *n* is the number of proteins defining the protein set; *v*_*i*_ are the protein signs (active or inactive) according to each disease/comorbidity definitions; and *y*_*i*_ are the resulting modeled signal values achieved by each protein “*i*” after stimulating the model with the corresponding drug.

#### Physiologically Based Pharmacokinetic Models

**Figure 5:**
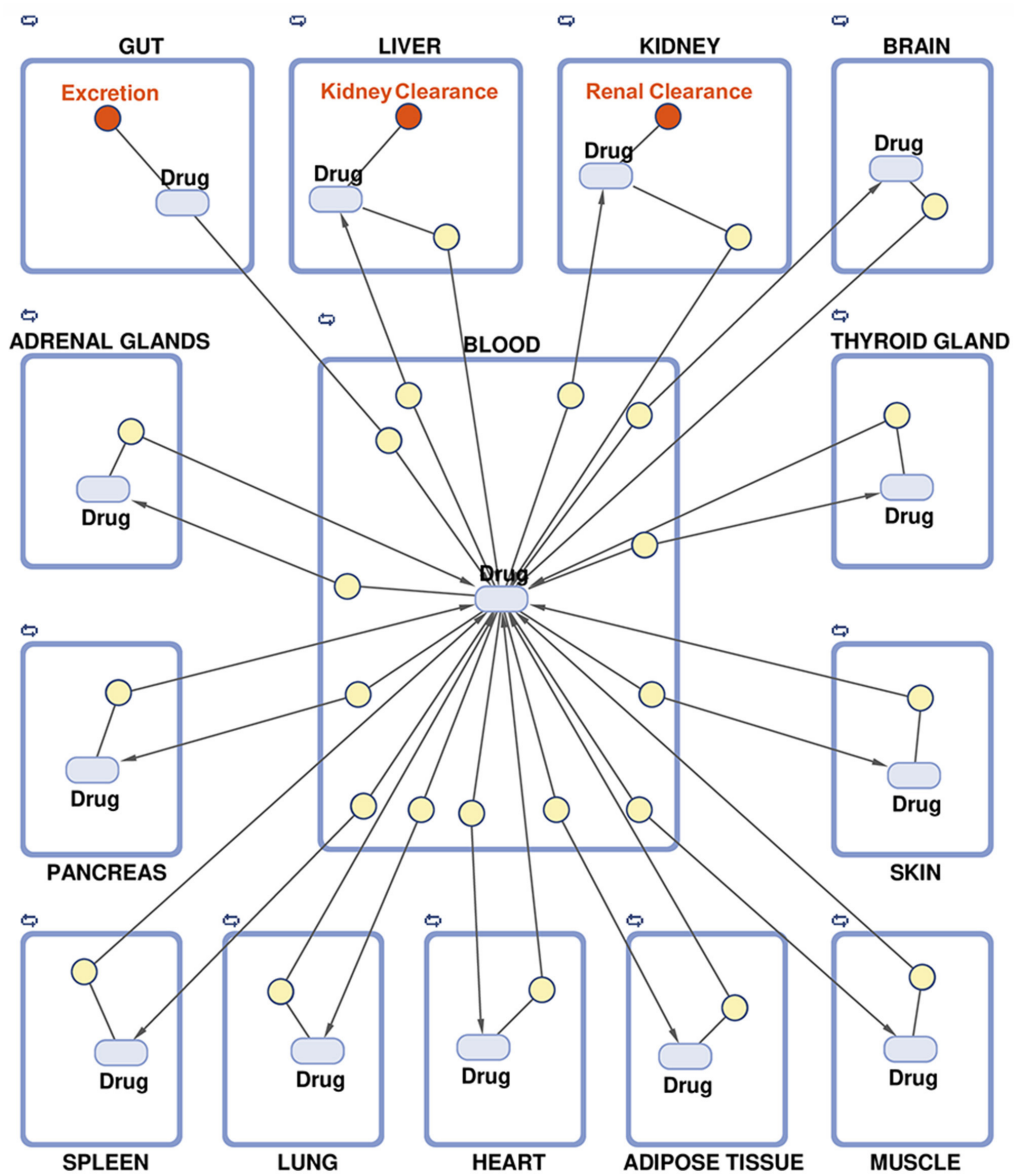
Schematic representation of the multi-compartment model for physiologically based pharmacokinetic modeling.

A PBPK model per virtual patient was built to describe the relationship between drug doses and drug concentration in different organs within the human body. The PBPK model structure used consists of 14 predefined compartments representing the human body's main organs and tissues, a simplified version of a previously reported model (72) (Figure 5). Blood acts as the central compartment by interconnecting the rest of the system through blood flows, and the whole system can be disturbed by administering a drug dose in any of the following organs or compartments: gut (oral drugs), blood (intravenous drugs), or skin (subcutaneous drugs). Similarly, clearance of drugs and compounds is restricted to three compartments: gut, liver, and kidneys. The equations associated with blood flow rates and organ/tissue volumes are taken from Brochot and Quindroit (73). These variables depend on cardiac frequency, age, BMI, and gender and yield individualized models as described elsewhere (74). Here, blood volume was readjusted to fit the volume of distribution of each compound for optimized modeling.

Parameters related to the anatomy and physiology of each specific patient's human body were used to mathematically describe drugs' internal flow, i.e., drugs' absorption, distribution, metabolism, and excretion (ADME) processes (75). The drug's absorption and clearance constant parameters were calculated by fitting the general model to existing real pharmacokinetics data points for d-amphetamine (d-Amph, active compound for LDX) and MPH (Table 2) (66–69). For other ADHD drugs (see Supplementary Methods in Supplementary Material 1), pharmacokinetics data used can be found in Supplementary Table D in Supplementary Material 2. Regarding MPH, as extended-release capsules are not easily simulated, an approximation using repeated administration of lower doses was used. This strategy had already been described for the two MPH extended-release formulations used here, and resulted in similar concentration dynamics: (i) for Medikinet^®^ with modified-release, considered to have an equivalent MPH bioavailability to Ritalin^®^ (56), a twice-a-day administration was simulated with half the dosage for each simulated administration, and (ii) for Concerta^®^, three administrations were simulated with one-third of the original dose for each administration (76).

The whole PBPK compartments model is implemented in MATLAB™, and differential equations describing the kinetics of the compounds and the fitting procedures are integrated by using SimBiology Toolkit (77).

#### Quantitative Systems Pharmacology Models—Quantitative Mechanism of Action

A QSP model enclosing PBPK model outputs and TPMS model maps was generated for each patient of the vPOPs. QSP models are generated following the TPMS methodology previously described (36) but incorporating drug concentration data at different timepoints in addition to molecular inputs, which add patient-specific quantitative data. To this end, a set of drug concentration timepoints in the target tissue—brain in this study—can be associated with the modulation of the drug's target proteins. Additionally, by applying the EC50 equation definition and using clinical efficacy observations, the drug's effect on the disease-characterized proteins in the target tissue can also be calculated (see Supplementary Methods in Supplementary Material 1). Accordingly, the resulting MPH and LDX drug's target modulation-efficacy relationships were used as extra parameters in the TPMS training set, resulting in the final QSP models. The latter had the same output format as the systems biology MoA models previously described (36), but included quantitative information related to drug concentration. Hence, these models were used to answer additional questions related to individual differences among patients or treatment comparisons. At least 50 mathematical solutions per patient were computed during the QSP modeling to account for intra-patient variability, with accuracies >85% with respect to the TPMS training set (36).

### Efficacy Outcomes and Measures Definition and Optimization

#### Molecular Measures

Due to the systems-biology–based nature of the virtual patients' resulting models, all measures were centered on protein activity. As previously described (36), after modeling a drug MoA on each patient, a protein activity value in the range (−1, 1) was obtained. These values can be either analyzed individually or combined in protein functional groups to evaluate biological concepts, such as diseases or comorbidities.

#### Efficacy Outcome

As for any clinical trial, in which the primary outcome is usually related to the drug's efficacy, our primary case-study goal was to identify and compare both drug's efficacies. Accordingly, a selection and conversion methodology were defined to select the protein set within the ADHD definition that best explained a chosen efficacy metric, and we transformed the protein activities of that set into a model-derived measure that correlated with an actual clinical measure. The clinical variable used here was the ADHD Rating Scale IV (ADHD-RS IV, change from baseline). Three steps were followed to convert TPMS-model protein activities into ADHD-RS IV values: (i) select a model-derived activity measure (i.e., tSignal) that could be used as a proxy for efficacy; (ii) carry out ADHD molecular characterization, which consisted on a curated review of the scientific literature available in the PubMed database to identify proteins functionally involved in ADHD (see Supplementary Methods in Supplementary Material 1; and Supplementary Tables A, B in Supplementary Material 2; and (iii) optimize by trimming the ADHD molecular definition using real clinical trial efficacy observations (using ADHD-RS IV). In the third step, a series of eligible ADHD clinical trials meeting our inclusion criteria and measuring ADHD-RS IV in relevant drugs (Table 3; Supplementary Methods in Supplementary Material 1) were compiled. The reported ADHD-RS IV values were then used for ADHD molecular definition refinement through Pearson's correlation (Supplementary Methods in Supplementary Material 1); the final ADHD definitions used for outcome measurement are displayed in Supplementary Table E in Supplementary Material 2.

**Table 3:** List of clinical trials used for attention-deficit/hyperactivity disorder model-derived efficacy measure optimization.

**Clinical trial number/PMID**	**Title**	**References**
**Adult clinical trials**		
PMID: 17137560	Efficacy and safety of dexmethylphenidate extended-release capsules in adults with attention-deficit/hyperactivity disorder	(78)
PMID: 20576091	Randomized, double-blind, placebo-controlled, crossover study of the efficacy and safety of lisdexamfetamine dimesylate in adults with attention-deficit/hyperactivity disorder: novel findings using a simulated adult workplace environment design	(79)
NCT00337285	A long-term, open-label, and single-arm study of NRP104 30, 50, or 70 mg per day in adults with attention deficit hyperactivity disorder (ADHD)	(33)
NCT01270555	Efficacy of bupropion SR for attention deficit hyperactivity disorder (ADHD) in adults with recent past or current substance use disorders	(80)
NCT01259492	A 40-week, randomized, double-blind, placebo-controlled, multicenter efficacy and safety study of methylphenidate HCl extended release in the treatment of adult patients with childhood-onset ADHD	(81)
NCT02141113	Double-blind, randomized, placebo-controlled, single- center, dose optimization study evaluating efficacy and safety of guanfacine hydrochloride in combination with Psychostimulants in adults aged 18–65 years with a diagnosis of ADHD	(82)
NCT02604407	A phase 3, randomized, double-blind, multicenter, placebo-controlled, forced-dose titration, safety and efficacy study of SHP465 in adults aged 18–55 years with attention-deficit/hyperactivity disorder (ADHD)	(83)
**Pediatric-adolescent clinical trials**		
NCT00507065	A phase III, randomized, multicenter, double-blind, parallel-group, placebo-controlled safety and efficacy study of ADDERALL XR with an open label extension, in the treatment of adolescents aged 13–17 with ADHD	(84)
PMID: 17577466	Efficacy and tolerability of lisdexamfetamine dimesylate (NRP-104) in children with attention-deficit/hyperactivity disorder: a phase III, multicenter, randomized, double-blind, forced-dose, parallel-group study	(85)
NCT00447278	A study comparing the effect of atomoxetine vs. other standard care therapy on the long term functioning in attention-deficit/hyperactivity disorder (ADHD) children and adolescents (ADHD LIFE)	(86)
NCT00393042	Sleep and tolerability of extended release dexmethylphenidate vs. mixed amphetamine salts: a double blind, placebo controlled study (SAT STUDY)	(87)
PMID: 21241954	Clonidine extended-release tablets for pediatric patients with attention-deficit/hyperactivity disorder	(88)
NCT00763971	A phase III, randomized, double-blind, multicentre, parallel-group, placebo- and active-controlled, dose-optimization safety and efficacy study of lisdexamfetamine dimesylate (LDX) in children and adolescents aged 6–17 with attention-deficit/hyperactivity disorder (ADHD)	(30)
NCT01244490	A phase 3, randomized, double-blind, multicentre, parallel-group, placebo- and active-reference, dose-optimization efficacy and safety study of extended-release guanfacine hydrochloride in children and adolescents aged 6–17 years with attention-deficit/hyperactivity disorder	(89)
NCT01328756	A phase 4, open-label, multicentre, safety study of lisdexamfetamine dimesylate in children and adolescents with attention-deficit/hyperactivity disorder (ADHD)	(90)

Model-derived ADHD outcome measures were optimized separately for adults' and pediatric-adolescent's clinical trials to reduce noise on the molecular definition.

### Data Analysis

For the analysis of the population demographic and PBPK parameters, descriptive statistics were used (mean and standard deviation, frequency tables, or pie charts), and appropriate parametric and non-parametric tests applied. The *p*-value was taken as a measure of the significance of the fitting to the reference population.

The data was analyzed employing MATLAB™ functions and Python or R packages to compare means and/or standard deviation between data distributions. Analyses with <30 samples were treated with non-parametric tests, while comparisons involving more than 30 samples were performed assuming a normal distribution and treated with parametric tests; in all cases, the applied test was reported. The statistical significance level was set at *p* < 0.05. False discovery rate (FDR) was used to control type I errors by applying the Benjamini-Hochberg (91) multi-test correction method, whenever relevant. All analyses were performed according to the described analytical strategy.

The accuracies of systems biology and QSP models were calculated for each solution within each individual model and expressed as the percentage of compliance of all drug-pathophysiology relationships included in the training set (36).

To evaluate the sensitivity of systems biology models, a local sensitivity analysis based in the SOBOL methodology (92) was performed to explore whether the variation in the protein activity (−1, 1) of the proteins in the models influenced the MoA models response of the two drugs (ADHD, as defined in Supplementary Tables A, B in Supplementary Material 2). According to the SOBOL terminology, TPMS models could be redefined as:


(2) $$\begin{array}{r} tSignal\left(ADHD\right)=\;TPMS\left(X\right)for\;X=\left\{X_{1},X_{2},X_{3},\ldots ,X_{n}\right\} \end{array}$$

Where *X*_*i*_ corresponds to each one of the parameters (here protein nodes activity) used in the models. Then, the variation of response model tSignal for each *X*_*i*_ parameter variation can be expressed as:


(3) $$\begin{array}{r} \frac{dTPMS}{d\left(X_{i}\right)}=\frac{d\left(tSignal\right)}{d\left(X_{i}\right)} \end{array}$$

The tSignal difference compared to the original model was computed for all values in the range tested, and the mean for each protein was calculated and evaluated as a percentage with respect to the maximal possible tSignal variation, set as 2 [(−1, 1) difference] minus the original tSignal.

An unsupervised clustering strategy was applied to obtain groups of two to seven clusters of MoAs to evaluate the molecular variability of the generated models. The two (adults and children-adolescents) complete sets of 1,000 QSP ADHD patient mechanistic models (500 for LDX and 500 for MPH) were evaluated separately, taking into account the final activation values of the ADHD protein effectors modulated by both drugs. Clusters were obtained using K means algorithm (93). The clustering analysis was performed using all features (effector proteins) and principal component analysis (PCA) dimensionality reduction with five dimensions (94). Four quality indicators were used to select the optimal number of clusters: Hopkins statistics (95) to measure the cluster tendency of a data set; Silhouette index (96) to weigh the cohesion of the clusters and Jaccard Bootstrap Index (97) to gauge the similarity and diversity of sample sets. Clusters were also filtered by heavily unbalanced groups, according to the Silhouette index ratio (96). Classification analysis, as described elsewhere (36), were applied to molecularly describe the identified clusters.

### Ethics

Only aggregated patient data from published clinical trials were used in the current project (30, 32, 33, 78–90, 98). Aggregated patient data prevents individual patients' identification and, thus, avoids the need for approval from an ethics committee or institutional review board.

### Computational Availability

All simulations described in this project were executed in the Anaxomics' cloud computing, which integrates more than 800 computational threads in machines with 64 Gigabytes of RAM. Software, databases, and tools are the property of Anaxomics Biotech.

## Results

### Demographic Characteristics

**Figure 6:**
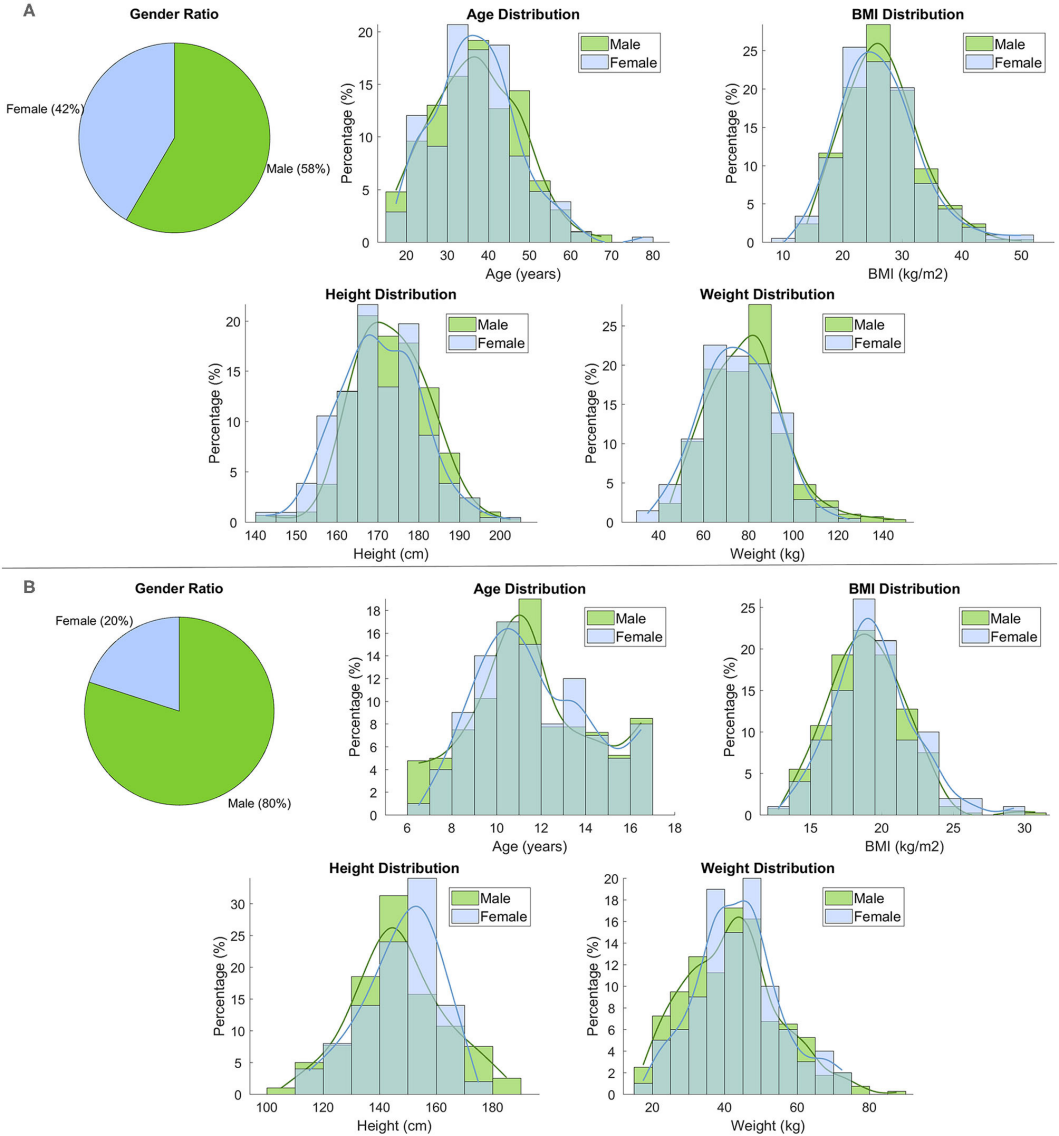
Demographic characteristics (sex, age, BMI, height, and weight) of **(A)** the adult virtual population (*N* = 500) and **(B)** the pediatric-adolescent virtual population (*N* = 500).

The characteristics of adult and pediatric-adolescent vPOPs of ADHD patients were generated from the proportions of demographic characteristics reported in the corresponding clinical trials. Additionally, eight subpopulations with different comorbidities (depression, anxiety, bipolar disorder, tics, and binge eating disorder) were created using the same method for both populations to evaluate the impact of comorbidities on the drugs' efficacy. The characteristics of our modeled vPOPs can be found in Figure 6A for adults and Figure 6B for the pediatric-adolescent population. The characteristics of the adult vPOP showed no significant differences with real reference populations extracted from clinical trials (Table 4). The same was true for the pediatric-adolescent vPOP (Table 5).

**Table 4:** Demographic characteristics of the adult virtual population and the reference population.

	**Virtual population** **(*N* = 500)**	**Reference population** **(*N* = 511)**	***p*-value^a^**
Sex (% females)	41.6	41.6	NA
Age (years)	36.98 ± 10.31	36.58 ± 10.10	0.37
Height (cm)	172.03 ± 9.98	171.7 ± 9.4	0.43
Weight (kg)	77.74 ± 16.95	78.75 ± 17.20	0.19
BMI (kg/m^2^)	26.51 ± 6.55	NA^b^	NA^b^

*Figures are mean ± standard deviation unless otherwise stated*.

*BMI, Body mass index; NA, not applicable*.

a *Calculated with the unpaired two-tailed T Student's test*.

b *For demographic data not provided in the reference clinical trial, European mean values were used*.

**Table 5:** Demographic characteristics of the pediatric-adolescent virtual population and the reference population.

	**Virtual population** **(*N* = 500)**	**Reference population** **(*N* = 111)**	***p*-value^a^**
Sex (% females)	20.0	20.0	NA
Age (years)	11.11 ± 2.73	10.90 ± 2.80	0.09
Height (cm)	147 ± 15.92	NA^b^	NA^b^
Weight (kg)	42.41 ± 12.70	43.60 ± 15.10	0.08
BMI (kg/m^2^)	19.1 ± 2.7	19.1 ± 3.4	0.81

*Figures are mean ± standard deviation unless otherwise stated*.

*BMI, Body mass index; NA, not applicable*.

a *Calculated with the unpaired two-tailed T Student's test*.

b *For demographic data not provided in the reference clinical trial, European mean values were used*.

### Local Sensitivity Analysis of Systems Biology-Based Models

TPMS-derived MoA models were subjected to sensitivity SOBOL analysis to evaluate whether the variation of molecular parameters would affect the models' response and to identify key molecules. The sensitivity evaluation was carried out for a range of values (−1, 1) for each protein. Although these models have about 5000 parameters, less than a third of them showed a real impact (difference >15%) on the output, which was less notorious in MPH (max difference ~17%) than in LDX (max difference ~32%) (Supplementary Table F in Supplementary Material 2). Interestingly, from the 30 most sensitive proteins, some were shared between both mechanisms (namely, NFKB1, PRKCA, PRKCZ, TRAF6, and PRKCB).

### Physiologically Based Pharmacokinetic Models

**Figure 7:**
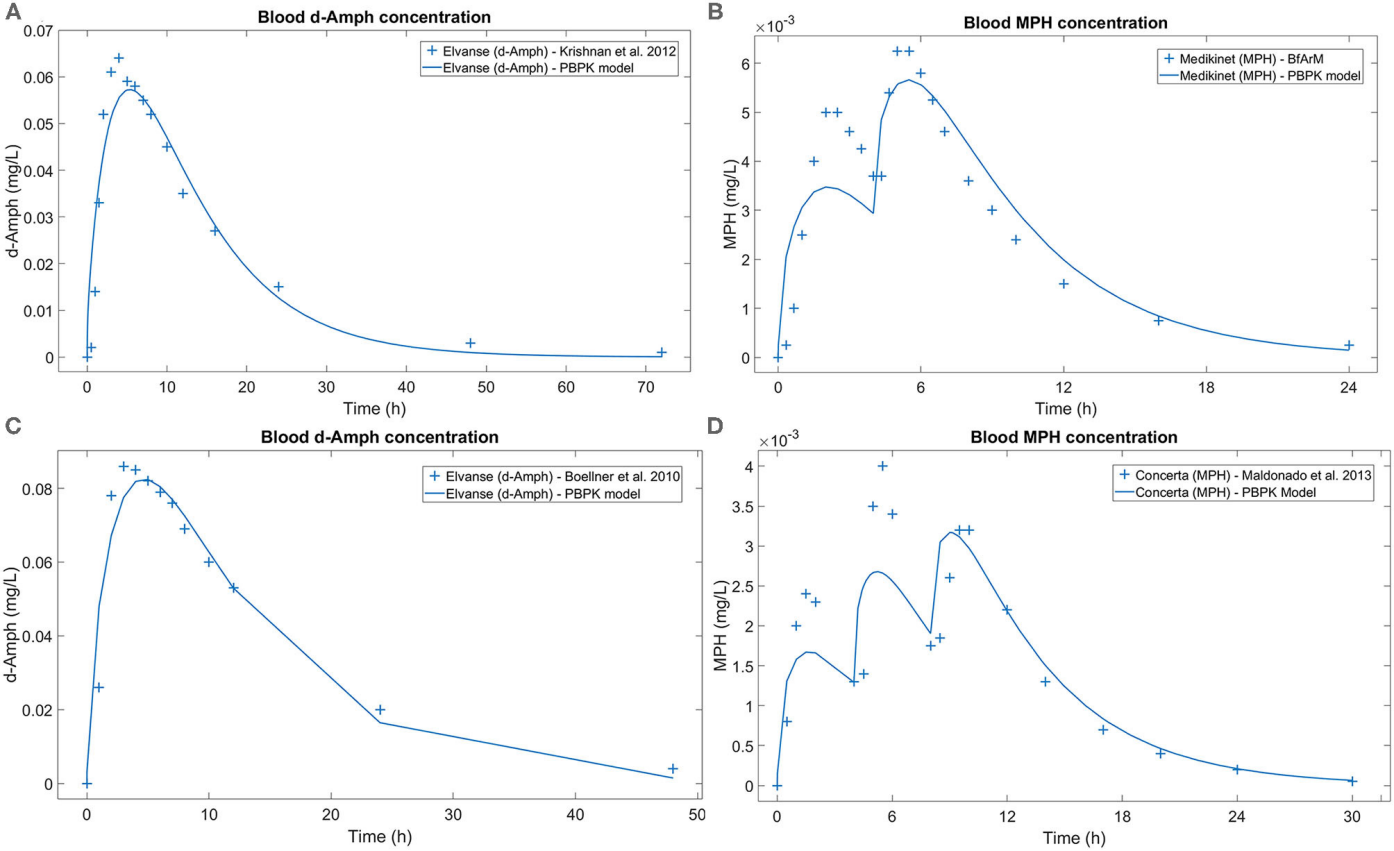
Blood d-Amph and MPH concentration comparison between real datapoints and the curve resulting from the PBPK model. **(A)** Generated for a standard adult patient after a single 70 mg dose of LDX, real datapoints obtained from Krishnan et al. (66); **(B)** generated for a standard adult patient after two 10 mg doses of MPH every 4 h, real datapoints obtained from BfArM (68); **(C)** generated for a standard pediatric patient after a single 50 mg dose of LDX, real datapoints obtained from Boellner et al. (67); and **(D)** model generated for a standard pediatric patient after three 5 mg doses of MPH every 4 h, real datapoints obtained from Maldonado et al. (69). d-Amph, d-Amphetamine; LDX, Lisdexamfetamine; MPH, Methylphenidate; PBPK, Physiologically based pharmacokinetic.

PBPK models simulating the available drug concentration in blood over time were obtained for LDX and MPH and for the two studied populations. Drug concentration models were fitted to real data resulting in similar blood drug concentration levels for a standard adult (male, 40 years old, 175 cm, 70 kg) and child (male, 8 years old, 30 kg, 130 cm) (Figure 7). PBPK model simulations complied with the observed *in vivo* curves, even for the case of MPH in children and adults, where approximating repeated administration of lower doses was required to model the modified-release formulations.

### Efficacy Outcomes and Measures Definition and Optimization

After the process of optimizing by trimming, 83 proteins (out of 86) were included in the pediatric-adolescent ADHD definition (ρ = −0.81) and 66 proteins (out of 86) in the adult ADHD definition (ρ = −0.79). The resulting molecular definitions found after optimizing the model-derived efficacy measures for each conditions' clinical efficacy can be found in Supplementary Table G in Supplementary Material 2. The subsequent regression lines, as well as the different study points used, are represented in Supplementary Figures A, B in Supplementary Material 1.

### Quantitative Systems Pharmacology Models in the Virtual Populations

The MoA of LDX and MPH in our populations of interest, inferred from QSP models, were obtained by combining PBPK models of the dosing schemes of these drugs and TPMS technology, which modeled the MoA of both drugs in ADHD. The simulation analyzed the whole available data on pathologies, drugs, and the population. The mean accuracy values obtained in mechanistic models for ADHD virtual patients were: 91.63% (adults treated with LDX), 91.71% (adults treated with MPH), 91.68% (children-adolescents treated with LDX), and 91.69% (children-adolescents treated with MPH). Thus, for each patient, activation/inhibition patterns of all proteins associated with the MoA of LDX and MPH were obtained. Drugs' efficacy on ADHD measured over each virtual patient was exclusively estimated using the above mentioned tSignal formula (Equation 1), which summed up the activity values of ADHD effector proteins. The tSignal formula was applied to the list of ADHD effector proteins optimized to fit clinical observations and provided high accuracy QSP models for the whole set of 1,300 patients comprising adults and children.

**Figure 8:**
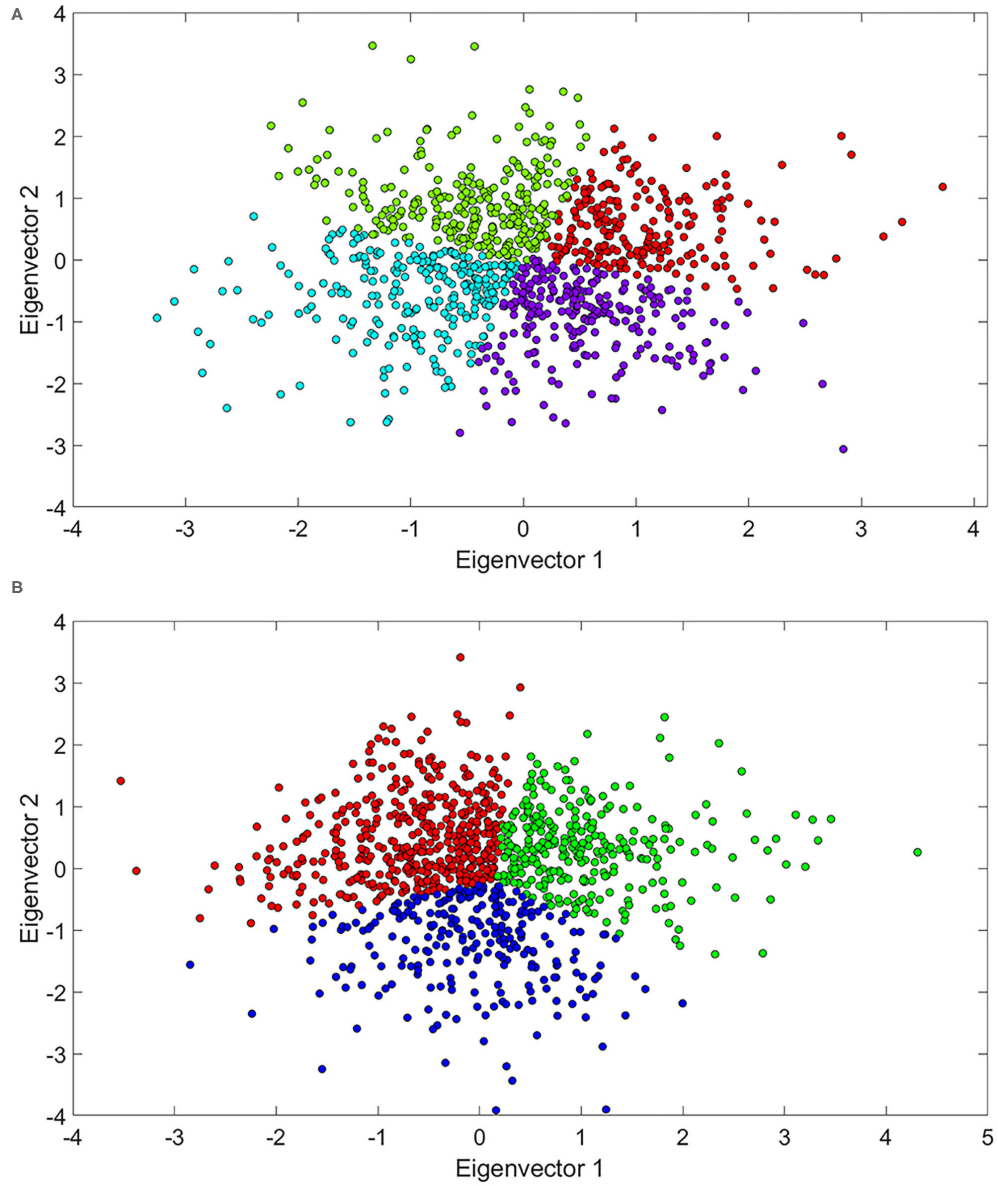
PCA representation, based on the modulation of ADHD effectors, of LDX and MPH mechanisms of action in **(A)** ADHD adult patients and **(B)** ADHD children-adolescent patients. ADHD, Attention-deficit/hyperactivity disorder; LDX, Lisdexamfetamine; MPH, Methylphenidate; PCA, Principal component analysis.

The ADHD population was subjected to clustering analysis to explore molecular variability within the LDX and MPH mechanistic models. The optimal number of clusters for adults was four different clusters, whereas three main clusters were identified for children, according to Hopkins statistics (0.82 and 0.89, respectively), Silhouette index (0.31 and 0.33, respectively), and Jaccard Bootstrap index (0.52 and 0.57, respectively). These results reflected drug-independent patient intrinsic variability since they clustered in a non–drug-dependent manner (Table 6). Clusters were represented using the two main components of PCA (Figure 8), which explained 66.7 and 12.4% of the observed variability in adults, and 61.4 and 19.4% in children-adolescents, respectively. The five most relevant proteins in the PC1 (eigenvector 1) of each population were – IL4, AKT3, NTRK2, IL5, and NTF3 for adults and – CRY1, AKT3, CRY2, AKT1, and AKT2 for children-adolescents.

**Table 6:** Distribution of LDX and MPH mechanistic models in the generated clusters.

**Drug**	**Cluster 1 (Red)**	**Cluster 2 (Green)**	**Cluster 3 (Blue)**	**Cluster 4 (Purple)**
**Adult models clustering**				
LDX	122	123	150	105
MPH	98	154	106	142
**Drug**	**Cluster 1 (Red)**	**Cluster 2 (Green)**	**Cluster 3 (Blue)**	
**Pediatric-adolescent models clustering**				
LDX	230	179	91	
MPH	215	118	167	

*LDX, Lisdexamfetamine; MPH, Methylphenidate*.

We also found that clustering was associated to differences in treatment efficacy in adults (ANOVA *p*-value = 2.515e-08) and children-adolescents (ANOVA *p*-value = 1.194e-09). In adults, cluster 4 showed the highest mean tSignal (*p*-value = 2.263e-09), while cluster 2 was the one presenting the lowest (*p*-value = 6.835e-04). In children-adolescents, the tSignal of cluster 1 was significantly higher (*p*-value = 2.752e-05) and that of cluster 3 was significantly lower (*p*-value = 7.397e-04) than the rest (Student's *T*-test). To further characterize the clusters, we performed an ANOVA analysis to identify potential differences on the demographic characteristics within the clusters. In the overall analysis, only weight was significantly different (*p*-value < 0.05) in adults (ANOVA *p*-value = 0.019), and no differences were found in children-adolescents. When comparing each cluster against the rest in adults, we found that BMI, weight, and gender ratio were significantly lower in cluster 4, while weight was slightly higher in cluster 2. In children- adolescents, BMI and weight were significantly lower in cluster 1, while weight was slightly higher in cluster 3 (Table 7).

**Table 7:** Results of the comparison analysis between demographic characteristics within the clusters.

**ID**	**Age, years**	**Height, cm**	**Weight, kg**	**BMI, kg/m^**2**^**	**Gender,** **M:F ratio**
**Adults**					
1	36.49 ± 9.96 (0.424)	172.19 ± 10.62 (0.797)	78.79 ± 17.67 (0.297)	26.85 ± 6.82 (0.387)	0.63 (0.141)
2	37.16 ± 10.73 (0.739)	172.02 ± 9.94 (0.979)	79.58 ± 16.95 (**0.034**)	27.02 ± 6.04 (0.131)	0.60 (0.454)
3	36.20 ± 9.75 (0.158)	171.80 ± 9.92 (0.659)	77.33 ± 15.73 (0.651)	26.53 ± 6.67 (0.953)	0.59 (0.826)
4	38.04 ± 10.73 (0.064)	172.16 ± 10.01 (0.820)	75.17 ± 17.23 (**0.006**)	25.62 ± 6.67 (**0.014**)	0.52 (**0.016**)
**Children-adolescents**					
1	10.95 ± 2.82 (0.095)	145.96 ± 16.13 (0.064)	41.42 ± 12.60 (**0.026**)	18.93 ± 2.69 (**0.031**)	0.81 (0.634)
2	11.22 ± 2.57 (0.431)	147.44 ± 14.53 (0.568)	42.70 ± 12.00 (0.646)	19.25 ± 2.71 (0.400)	0.76 (0.067)
3	11.27 ± 2.75 (0.283)	148.29 ± 16.96 (0.131)	43.80 ± 13.51 (**0.041**)	19.36 ± 2.68 (0.118)	0.83 (0.170)

Figures are mean ± standard deviation (and p-values*).

^*^T-student-test: each cluster vs. rest of clusters.

*ANOVA, Analysis of variance; BMI, Body mass index; ID, Cluster ID; M:F ratio, Male to female ratio*.

*p-values in bold are those considered significant (p <0.05)*.

## Discussion

Herein, the technology to create populations of virtual patients and the subsequent ISCT is described in the case-study of LDX and MPH head-to-head comparison in the context of ADHD treatment. Adult and pediatric-adolescent vPOPs were obtained, and PBPK and QSP models were generated successfully to provide the basis for identifying mechanistic differences between the two drugs, patient cohort differences, inter- and intra-patient response variability.

Preliminary evaluation of the models revealed some insights on the factors affecting MoA-related treatment efficacy. The sensitivity analysis of systems biology MoA models provided a list of common proteins that might affect both drugs' efficacy: proteins involved in the NF-κB signaling pathway (NFKB1 and TRAF6) and PKC (alpha, beta, and zeta types). The pleiotropic nature of these proteins and their involvement in several signaling processes could explain their potential impact in the sensitivity of mechanistic models. However, a detailed evaluation of each drug mechanistic model should provide further knowledge on the key proteins involved.

QSP model clustering analysis indicated the presence of several response patterns, not clearly defined by drug treatment. The protein activity-based unsupervised clustering was somehow associated to response level, and PCA analysis revealed some relevant proteins that could be exerting this effect, including: dopamine signaling-related AKT proteins (99), neurotrophins related to neural viability and dopamine regulation in ADHD (100–103), circadian rhythm proteins related to ADHD and comorbidities–associated sleep disturbances (104–106), and cytokines related to neuroinflammation and Th2 response and ADHD (107–110). Regarding clinical characteristics, a possible correlation was found between lower weight, female gender, and lower BMI, with a higher tSignal or better efficacy in adult population. Similar results were found in the children-adolescent population, were higher tSignals were found in the group with lower weight and BMI. In this sense, previous reports had already suggested a relationship between drug efficacy and BMI (111).

### Related Work

Virtual populations have been generated in the past to assist in solving complex medical issues. The FDA has accepted a type 1 diabetes simulator to replace animal testing in pre-clinical trials (112). Besides, *in silico* cloning of data from individual type 1 diabetes patients to improve algorithms for closed-loop insulin delivery systems has been reported in 12 and 47 virtual patients in studies that aimed to tackle the challenging problem of inter- and intra-subject variability (113, 114). Likewise, a virtual population of 50 individuals has been generated to test *in silico* drug cardiotoxicity and account for inter-subject variability in clinical studies with toxicological endpoints (115). Such approaches were warranted considering the high variability of the evaluated pharmacokinetic parameters in a short time. However, they were limited in the number of virtual patients that could be generated accurately. Therefore, considering that ADHD is not as varying in brief periods and that such pharmacokinetic detail was unnecessary, a higher number of patients could be generated in our study. On the other hand, a multi-compartment model with a large virtual population size has been published on trauma-induced critical illness that showed how the molecular and cellular events taken as a whole could manifest heterogeneously on individuals (116). These results were in agreement with ours, which showed different clusters of patients that could correspond to different response profiles to a certain point, independent from drug treatment.

Virtual populations combined with PBPK modeling have been used successfully to predict the pharmacokinetic profile of a drug and evaluate potential drug-drug interactions for a specific ethnicity (117). In addition, a PBPK model combined with systems-biology techniques has been reported and validated as an efficient tool for assessing risk exposure to certain volatile organic compounds (118). Furthermore, multi-compartment QSP has been used to model immunotherapies in breast cancer (119). When associated with pharmacokinetics and pharmacodynamics data, it has been reported in an *in silico* virtual clinical trial to analyze predictive biomarkers in certain breast cancers (120). Hence, PBPK and QSP models have been established as powerful computational tools for *in silico* simulations.

Finally, only a few *in silico* head-to-head trials have been published. A recent study compared two insulin therapies for type 1 diabetes treatment by using the abovementioned FDA-approved simulator and pharmacokinetics models to compare two designs, crossover and parallel (121). The parallel design was justified because it would likely be preferred in a real setting for practical reasons, which is not necessarily true in the case of our study on ADHD. Another head-to-head mechanistic study comparing two lung cancer treatments has been reported by our group, whereby a similar approach to the one here described was undertaken (122). However, our previous study did not require generating virtual populations nor used PBPK or QSP models to reach its conclusions.

These examples of application of *in silico* modeling approaches in different therapeutic areas bear witness to an increasing tendency to use newly available high performance computing technologies in the field of biomedicine. The use of these technologies will help advancing toward the implementation of precision medicine pipelines and personalizing the healthcare provided to patients.

### Strengths and Limitations

TPMS models are constructed considering the whole human protein network and a wide range of drug-pathology relationships, not only limited to ADHD or psychiatric ailments, which, in part, attenuates the potential bias on information regarding drugs or disease of interest. As defined by Jorba et al. (36), only MoA models with accuracies above 85% against the training set were used to ensure good quality and general extrapolation of results. This systems biology-based methodology has been reported to be successful, with results validated by *in vitro* and/or *in vivo* models (123–125).

Only limited by computational power, ISCT allows enrolling a large number of patients with several neurophysiological ADHD subtypes, which can be difficult, costly, and even not feasible in a conventional clinical trial setting. Virtual patients generated in our study were defined by the drugs' molecular mechanisms, allowing the exploration of the complete clinical and molecular landscape of each patient. Furthermore, our ISCT design had a large enough sample size and considered pools of mathematical solutions for each patient—instead of a single mathematical model per patient—which ensured that the simulations were robust and appropriate for data analysis.

However, our study presented some limitations. Firstly, our models depended on the current knowledge of human physiology, particularly on the drugs and disease under investigation, as well as protein interactions and pathways described and involved in the MoA. Therefore, our models could have been susceptible to missing data, errors, and bias, and some aspects could have been overlooked. For instance, unknown targets or yet undescribed pathophysiological ADHD processes might play a role in the MoA of the evaluated drugs. ADHD and its associated comorbid psychiatric disorders present a high genetic and signaling overlap (126, 127), which could act as confounding factors at the clinical and molecular levels. Accordingly, the molecular characterizations used for modeling could be biased; prospective data could expand the knowledge on these diseases and, therefore, improve our model-derived conclusions.

Secondly, our approach considered only the impact of demographic characteristics on the PBPK modeling (i.e., drugs' absorption, distribution, metabolism, and excretion). However, other consequences of these characteristics at the ADHD pathophysiology level were not considered, because of the absence of (i) clear molecular information to include in the ADHD definitions for each patient profile, and (ii) reliable sources of information to properly model these characteristics at the molecular level. This limitation could prevent the modeling and detection of relevant results regarding these characteristics, such as age-related neurodevelopment (128, 129), differences between children, adolescent and adults (130, 131) or the potential role of sex-dependent differences (132–134). Future data on large sets of patients, or specific research on the impact of those characteristics on ADHD, might allow to improve our models and derived conclusions.

Thirdly, all mathematical models are subjected to the limitation of not being able to fit 100% the training data information. In our approach, while we obtained a pediatric virtual population with demographic values non-significantly different from the reference clinical trial population, the obtained *p*-values for age and weight were close to the significance threshold. These parameters proved to be more difficult to fit in pediatric than in adult virtual populations. While clinical trials only report average weight and age, general pediatric population weight distributions obtained from growth information (35) are age-dependent. Accordingly, setting a higher threshold of significance (*p* > 0.1 or even *p* > 0.2) during the randomization procedure might ensure obtaining a fitter population, especially regarding the pediatric case. In this specific scenario, as the case-study objective for the generation of the ISCT was a head-to-head between LDX and MPH using the exact same population, this bias was not expected to significantly affect the results. TPMS-based models are not an exception either (36). Each virtual patient was constructed with at least 50 solutions, and a population sample size larger than the minimum calculated was used to dampen this effect. TPMS models present an inherent variability, rendering them useful to explore molecular variability within human physiology (36); through an adequate management of the model's variability and considering an appropriate sample size, the best solutions could be obtained.

Finally, our study's primary outcome was generated with information from literature on the drugs used for ADHD treatment and their measured clinical effect. The values used for the training process were the average values reported in those publications (Table 3). However, a great dispersion was observed. For instance, while the mean ADHD-RS IV value associated with amphetamine was −18.1, the authors report a range of response between −4.68 and −31.52, representing a 74% deviation from the mean, clearly higher than the dispersion values generated with our models (Supplementary Figure A in Supplementary Material 1). The dispersion identified in clinical trials was probably due to demographic and metabolic differences between patient cohorts and how the principal variable was measured. This effect appeared in all analyzed drugs, and we estimated an average dispersion of 57% for all of them. The dispersion in the efficacy measured from the clinical trials cannot be mathematically treated without accessing patient data, which is not available; at this point, we had to resort to a naïve pooled approach, risking its associated limitations. In such cases, the best approach to obtain a drug efficacy value is to compute the mean of the values reported by different authors. Selection bias can also induce errors when using external data. To attenuate its effect, clinical trials assessing a wide range of drugs were used in our study. On the other hand, another limitation associated to the outcome measure used would be of clinical nature; ADHD symptom scales are based on questionnaires to the patient or the physician, that comprise several aspects of a complex psychiatric disease. These clinical measures might not be as directly associated to molecular or biologically measurable factors (such as blood pressure when studying hypertension). To minimize both technical and clinical limitations of the outcome measure used, we selected the ADHD-RS IV scale as this was the scale with the largest amount of clinical trial information for different mechanisms of action, so the model efficacy measures could be properly optimized to fit clinical data. Our approach tried to compile the largest amount of available information around patients, disease, and treatments at the molecular and clinical level and provided benchmarks to validate the different steps of the study. Nonetheless, a corroboration of the herein described procedure to infer new actual clinical results with independent (existent or new) experiments is called for.

In our ISCT, most of these sources of error could translate into an error in the estimation of the principal variable, evaluated by the Pearson correlation coefficient (Supplementary Figures A, B in Supplementary Material 1). Interestingly, the Pearson correlation coefficients obtained after lineal regression adjustment for the adult and pediatric-adolescent populations were high given the large dispersion shown in values from clinical practice for the same drugs (Supplementary Table E in Supplementary Material 2).

## Conclusion

The methods here illustrated described the step-by-step process for creating a virtual population of patients treated with two drugs for ADHD management, LDX and MPH, with the aim of designing an ISCT for their comparison head-to-head. Our study provided adult and pediatric-adolescent vPOPs and generated QSP models to infer, after analysis, the MoA of these two drugs. This theoretical model, and its use for a head-to-head analysis, would allow obtaining conclusions classified as MEDIUM impact according to MID3 guidelines. Although experimental and clinical assays are warranted to validate or refute these potential results before translation into clinical practice, the mechanistic-driven modeling techniques used here should be accepted as hypothesis-generation solid tools with a remarkable ability to provide molecular detail. Besides, from a scientific evidence point of view, complementing meta-analyses with theoretical models, such as the ones here presented, can palliate the lack of costly, though necessary, head-to-head clinical trials. Altogether, *in silico* techniques can contribute to advancing the understanding of diseases' pathophysiology and the molecular MoA of available therapies, with the ultimate goal of reaching personalized medicine.

## Data Availability Statement

The original contributions presented in the study are included in the article/Supplementary Material, further inquiries can be directed to the corresponding authors.

## Author Contributions

JRG-C, JQ, VM, TP-R, CM, and JM conceived the study. JRG-C, JQ, GJ, and VJ performed the investigation and drafted the first version of the manuscript. GJ, VJ, BO, XD, and JM developed the methodology of the study. GJ, VJ, and JM undertook software related tasks. VM, TP-R, and CM managed the project and JRG-C, JQ, BO, XD, and JM supervised and validated it. GJ and VJ aided in visualization tasks. All authors reviewed and edited its final version.

## Funding

This study was funded by Takeda. Public funders provided support for some of the authors' salaries: GJ has received funding from the European Union's Horizon 2020 research and innovation program under the Marie Skłodowska-Curie Grant Agreement No. 765912. VJ is part of a project (COSMIC; www.cosmic-h2020.eu) that has received funding from the European Union's Horizon 2020 research and innovation program under the Marie Skłodowska-Curie Grant Agreement No. 765158.

## Conflict of Interest

JRG-C has served as speaker for Takeda and Shire and has received research funding from Shire. JQ has served as speaker and/or on scientific advisory boards for Takeda, Janssen, and Rubio. GJ, VJ, and JM are full-time employees at Anaxomics Biotech. VM, TP-R, and CM are full-time employees at Takeda. The remaining authors declare that the research was conducted in the absence of any commercial or financial relationships that could be construed as a potential conflict of interest.

## Publisher's Note

All claims expressed in this article are solely those of the authors and do not necessarily represent those of their affiliated organizations, or those of the publisher, the editors and the reviewers. Any product that may be evaluated in this article, or claim that may be made by its manufacturer, is not guaranteed or endorsed by the publisher.
