# Supplementary material for: Methods to Develop an in silico Clinical Trial: Computational Head-to-Head Comparison of Lisdexamfetamine and Methylphenidate
Source: Front Psychiatry. 2021 Nov 3;12:741170. doi: 10.3389/fpsyt.2021.741170 (PMC8595241; doi:10.3389/fpsyt.2021.741170)
Supplement: Supplementary file 1 [file Data_Sheet_1.pdf]

SUPPLEMENTARY METHODS

Methods to develop an in silico clinical trial: Computational head-to-head comparison of lisdexamfetamine and methylphenidate

José Ramón Gutiérrez-Casares [1†*], Javier Quintero[2†], Guillem Jorba [3,4†*], Valentin Junet[3,5], Vicente Martínez[6], Tamara Pozo-Rubio[6], Baldomero Oliva[4], Xavier Daura[5,7], José Manuel Mas[3], Carmen Montoto[6]

AFFILIATIONS: [1] Unidad Ambulatoria de Psiquiatría y Salud Mental de la Infancia, Niñez y Adolescencia. Hospital Perpetuo Socorro. Badajoz, Spain; [2] Servicio de Psiquiatría. Hospital Universitario Infanta Leonor. Universidad Complutense. Madrid, Spain; [3] Anaxomics Biotech, Barcelona, Spain; [4] Structural Bioinformatics (GRIB). Departament de Ciències Experimentals i de la Salut, Universitat Pompeu Fabra, Barcelona, Spain; [5] Institute of Biotechnology and Biomedicine, Universitat Autònoma de Barcelona, Cerdanyola del Vallès, Spain; [6] Medical Department. Takeda Farmacéutica España, Madrid, Spain; [7] Catalan Institution for Research and Advanced Studies (ICREA), Barcelona, Spain.

*** Correspondence:**
José Ramón Gutiérrez Casares
jrgutierrezcasares@gmail.com
Guillem Jorba

guillem.jorba@anaxomics.com

† These authors have contributed equally to this work and share first authorship.

Bibliography-based characterization and searches

Bibliography-based drug characterization

Aside from a review of official regulatory documentation and drug-target dedicated databases, a review of the currently available bibliography regarding known targets of the drugs was performed in PubMed on April 27, 2020. The specific searches performed were the following:

- ("Elvanse" [Title/abstract] OR "Vyvanse" [Title/abstract] OR "Lisdexamfetamine" [Title/Abstract]) AND ("molecular" [Title/Abstract] OR "mechanism" [Title/Abstract] OR "pathophysiology" [Title/Abstract] OR "pathogenesis" [Title/Abstract] OR "mode" [Title/Abstract] OR "action" [Title/Abstract] OR "signaling" [Title/Abstract] OR "signalling "[Title/Abstract] OR "expression" [Title/Abstract] OR "activation" [Title/Abstract] OR "inhibition" [Title/Abstract] OR "activity" [Title/Abstract])
- ("Methylphenidate" [Title] OR "Medikinet" [title/abstract] OR "Concerta" [title/abstract] OR "Medikinet"[title/abstract]) AND ("molecular" [Title] OR "mechanism" [Title] OR "pathophysiology" [Title] OR "pathogenesis" [Title] OR "action" [Title] OR "signaling" [Title] OR "signalling " [Title])

All articles were analyzed at the title and abstract level. The presence of molecular information was reviewed in depth to identify protein/gene candidates to be considered drug target candidates.

Bibliography-based conditions characterization

For disease characterization, we initiated an extensive and careful full-length review of relevant articles in the PubMed database (up to January 21, 2020) that included the following search strings:

- ADHD: ("Attention deficit hyperactivity disorder" [Title] OR "ADHD" [Title] OR "Attention-Deficit/Hyperactivity Disorder" [Title]) AND ("pathogenesis" [Title/Abstract] OR "pathophysiology" [Title/Abstract] OR "molecular" [Title/Abstract]) AND Review [ptyp].
- Depression: ("Depression" [Title] OR "Major Depressive Disorder" [Title]) AND ("Molecular" [Title/Abstract] AND ("Pathophysiology" [Title/Abstract]) OR ("Pathogenesis" [Title/Abstract]) AND (Review[ptyp] AND "2015/01/28" [PDat] : "2020/01/28" [PDat])
- Anxiety: ("Anxiety disorders" [Title] OR "Anxiety" [Title]) AND ("Pathogenesis" [Title] OR "Pathophysiology" [Title] OR "Molecular"[Title])
- Bipolar Disorder: ("Manic-Depressive" [Title] OR "Bipolar" [Title] OR "Manic Depressive" [Title] OR "Manic Disorder" [Title] OR "Manic Depression" [Title]) AND ("Pathogenesis" [Title/Abstract] OR "Pathophysiology" [Title/Abstract] OR "Molecular" [Title/Abstract]) AND Review[ptyp]

- Tics Disorder: (“TICS” [Title] OR "Tic Disorder" [Title] OR "Tic Disorders" [Title]) AND ("Pathogenesis" [Title/Abstract] OR "Pathophysiology" [Title/Abstract] OR "Molecular" [Title/Abstract])
- Binge eating: ("Binge Eating Disorder" [Title] OR "Binge Eating" [Title]) AND ("Pathogenesis" [Title/Abstract] OR "Pathophysiology" [Title/Abstract] OR "Molecular" [Title/Abstract])

The list of publications identified in the specific searches was retrieved and assessed at the title and abstract level. If molecular information describing pathophysiology conditions was found, the full texts were thoroughly reviewed to identify the main pathophysiological processes known to be involved in the diseases (Table A in the S2 File). Subsequently, each pathophysiological process was further characterized at the protein level by using the retrieved publications. Accordingly, proteins whose activity (or lack thereof) are functionally associated with the development of the condition were identified (Table B in the S2 File).

Clinical trial information compilation

To accurately obtain a model-derived efficacy value to fit clinical efficacy values, clinical trials that assess the efficacy of drugs currently approved and commonly used in clinical practice were retrieved from clinicaltrials.gov and PubMed. Only phase III clinical trials that were interventional (i.e. included at least an arm treated with the drug of interest), completed, and had published results were considered. Once listed, these clinical trials were evaluated to select the efficacy scale most frequently used and the best representative of drug variability (i.e. that included data from a vast number of drugs). Accordingly, the ADHD Rating Scale IV (ADHD-RS IV) and the following list of drugs were considered: Amphetamine, Atomoxetine, Bupropion, Clonidine, Dexmethylphenidate, Guanfacine, Lisdexamfetamine, and Methylphenidate. The selected drugs were characterized at the molecular target and pharmacokinetic levels (Table D in the S2 File).

Modelling methodology and algorithms

Virtual population modelling

Adult virtual populations (vPOPs) are created by assigning demographic variables (age, weight, height, and body mass index [BMI]) to virtually generated patients by using a modification of the algorithm proposed by Allen *et al*. (1). Accordingly, we define as *original population* the one whose characteristics we would like to mimic and as *standard population* the reference use to complete missing demographic characteristics. Firstly, a multivariate normal distribution (MVND) with the given means and standard deviations for each variable from the original population is created. When data about a specific parameter is not available, the information is

taken from the standard population distribution (in this study, for the adult population, a standard European distribution (2) was used). Since BMI, weight, and height are related through equation 1, only age and one pair of the morphometric parameters (weight and BMI, weight and height, or BMI and height) are generated.

$$BMI(kg/m^2) = \frac{weight(kg)}{height(m)^2} \qquad \text{equation [1]}$$

Secondly, a cost function based on the original population demographic parameters is used with the objective of being minimized until the generated population resembles the available information on the original population. A simulated annealing strategy is used to minimize the cost function by using as starting points the patients generated according to MVND values.

Let n be the number of patients to generate; μ_i, σ_i, m_i, M_i the mean, standard deviations, minimums, and maximums of the original population's age, height, weight, and BMI (i); X_i the n dimensional vector containing the variable i's values of the generated population; and $X_{i,j}$ the $n \times 2$ matrix containing the generated population's values of the variables i and j so that it represents the concatenation of X_i with X_j. For any data vector X generated with the multivariate distribution, let mean(X), std(X), min(X) and max(X) be the mean, standard deviation, minimum, and maximum of X. Two different cost functions are defined: the first one contains only age as a single item, and the second equation is based on equation 1, relating two of the morphometric parameters (BMI, weight, and height).

For age (a):

$$f_a(X_a) = (\text{mean}(X_a) - \mu_a)^6 + (\text{std}(X_a) - \sigma_a)^4 + \sum_{x_a \in X_a} \max\left(\left(x_a - \frac{m_a + M_a}{2}\right)^2 - \left(\frac{M_a - m_a}{2}\right)^2, 0\right) \quad \text{equation [2]}$$

For a pair of the morphometric parameters (BMI, weight, and height) (i, j):

$$f1_{i,j}(X_{i,j}) = (\text{mean}(X_i) - \mu_i)^6 + (\text{std}(X_i) - \sigma_i)^4 + (\min(X_i) - m_i)^2 + (\max(X_i) - M_i)^2 + (\text{mean}(X_j) - \mu_j)^6 + (\text{std}(X_j) - \sigma_j)^4 + (\min(X_j) - m_j)^2 + (\max(X_j) - M_j)^2 \quad \text{equation [3]}$$

Since i and j are generated independently from the remaining demographic variable (either BMI, weight, or height), and to ensure the latter stays in a plausible range, the cost function was extended by using equation 3, which also depends on k:

i = weight; j = BMI; k = height

$$f2.1_{i,j,k}(X_{i,j}) = \left(\text{mean}\left(\sqrt{\frac{X_i}{X_j}}\right) - \mu_k\right)^6 + \left(\text{std}\left(\sqrt{\frac{X_i}{X_j}}\right) - \sigma_k\right)^4 + \sum_{(x_i,x_j)\in X_{i,j}}\left(\max\left(\left(\sqrt{\frac{x_i}{x_j}} - \frac{m_k+M_k}{2}\right)^2 - \left(\frac{M_k-m_k}{2}\right)^2, 0\right)\right) \quad \text{equation [4]}$$

i = height; j = BMI; k = weight

$$f2.2_{i,j,k}(X_{i,j}) = \left(\text{mean}(X_i^2 \cdot X_j) - \mu_k\right)^6 + \left(\text{std}(X_i^2 \cdot X_j) - \sigma_k\right)^4 + \sum_{(x_i,x_j)\in X_{i,j}}\left(\max\left(\left(x_i^2 \cdot x_j - \frac{m_k+M_k}{2}\right)^2 - \left(\frac{M_k-m_k}{2}\right)^2, 0\right)\right) \quad \text{equation [5]}$$

i = height; j = weight; k = BMI

$$f2.3_{i,j,k}(X_{i,j}) = \left(\text{mean}\left(\frac{X_j}{{X_i}^2}\right) - \mu_k\right)^6 + \left(\text{std}\left(\frac{X_j}{{X_i}^2}\right) - \sigma_k\right)^4 + \sum_{(x_i,x_j)\in X_{i,j}}\left(\max\left(\left(\frac{x_j}{{x_i}^2} - \frac{m_k+M_k}{2}\right)^2 - \left(\frac{M_k-m_k}{2}\right)^2, 0\right)\right) \quad \text{equation [6]}$$

Where $\frac{X_i}{X_j}$ corresponds to the pointwise division, i.e. a vector where the lth element is the lth element of X_i divided by the lth element of X_j. Similarly, $X_i \cdot X_j$ and $\mathrm{X}^2 = X_i \cdot X_i$ are the pointwise multiplications. The final cost function equation for BMI/weight/height results as follows:

$$f_{i,j,k}(X_{i,j}) = f1_{i,j}(X_{i,j}) + f2_{i,j,k}(X_{i,j}) \quad \text{equation [7]}$$

A one-sample z-test (alpha= 0.05) is also used to ensure the new population preserves the original data distributions; otherwise, the population is recalculated.

In pediatric-adolescent virtual populations, and because morphometric measures drastically depend on age, a slightly different approach was undertaken. The first step was generating a sample population using the percentiles information reported by the World Health Organization in pediatric-adolescent populations (3) and by randomly generating plausible values setting the percentiles as probability density function points (4). Subsequently, a resampling strategy based on the Metropolis-Hastings method (5) was used to select a sub-population fulfilling the means and standard deviations of the original population. An initial sub-population set was chosen randomly. Then, an iterative process was carried out by continuously replacing patients from the sub-sample with the ones in the generated sample population. If the newly replaced patient resulted in a sub-population closer to the original target population, that population was chosen. Otherwise, the old patient remained in the population. This process continued until a p-value > 0.05 was obtained, according to a one sample z-test between distribution values of the sub-population and the original population.

Quantitative Systems Pharmacology models – Physiologically-based pharmacokinetic data integration in Therapeutic Performance Mapping System models

The training data used by Therapeutic Performance Mapping System (TPMS) models (Table C in S2 File) consists of physiologically known stimulus-response relationships (e.g. drug-indication) that must be achieved (6). Therefore, to integrate the patient-specific physiologically-based pharmacokinetic (PBPK) concentration data with TPMS resulting in the final Quantitative Systems Pharmacology (QSP) models, the individuals' drug concentration variation data need to (i) be transformed into a protein activity-like measure (stimulus); and (ii) be associated to a molecular effect (response). To that end, drug concentration is translated into drug target protein's grade of activation/inhibition and, in parallel, is related to the pathology—ADHD in this case—inhibition or reduction by using a set of equations based on the half-maximal response concentration (EC50) definition and clinical efficacy data.

To calculate the stimulus, the following procedure and equations are used. PBPK models describe the variation of drug concentration in the different compartments over time. Given a specific compartment, the drug concentration (C) variation over time can be expressed as a vector of drug concentration values for i timepoints (C_i). The drug concentration is then related to its protein targets (Table 1 in the main text). According to the TPMS definition, the activity of a protein P can be treated as a normalized vector with values in the range [-1,1], where 1 represents the maximal functional capability of the component to develop its activation functional role, -1 corresponds to the maximum inhibition capacity, and 0 represents the null capability of developing tasks. For each setting, and assuming that the maximal absolute value of P will be obtained when drug concentration is maximal ($\max(C)$), we define Sig as the protein sign (+1 when drug activates P, -1 for inhibition), and a vector of protein target activity over time i as:

$$\mathrm{P}_i = \mathrm{C}_i / \max(C) \times Sig \qquad \text{equation [8]}$$

Response values are also calculated by using drug concentration over time (C_i) but refer to the drug effect over the disease. The transformation into pathology response values can be obtained applying the concept of EC50, according to equation 9 (7), being Eff the effect of the drug over the pathology, which will range from 0 to 1:

$$Eff = \frac{\mathrm{C}}{EC50 + \mathrm{C}} \qquad \text{equation [9]}$$

As real drug's EC50 were not available, $EC50'$ was here defined as a model-derived proxy related to (clinical) efficacy or drug effect. Also, the resulting models' tSignals were used as a model-derived measure of the drug's impact on the pathology (Eff' i.e. the model equivalent to the parameter Eff). Then, to pre-calculate the $EC50'$ and be able to obtain the Eff', the theoretical

mechanism of action model between the drugs under study and the molecular descriptors of the pathology were built, as described by Jorba et al. (6), and the resulting tSignals extracted. The latter was assumed as a drug's maximal effect and, by applying equation 9, $EC50'$ could be defined as a function of the Eff' when the maximal drug concentration was achieved ($\max(C)$):

$$EC50' = \left(\frac{\max(C)}{\text{Eff}'} - \max(C)\right) \qquad \text{equation [10]}$$

To render $EC50'$ an estimate of clinical efficacy, it was weighted taking into account the real clinical efficacies ($clEff$) of the whole set of drugs to be considered in the study; thus, $EC50'$ was a parameter relative to the set of drug efficacies included in the analysis. For each drug, let $clEff$ be the clinical efficacy value; $\max(clEff)$ the maximum clinical efficacy found from the whole set of drugs considered; and Eff' the tSignal extracted from the TPMS model. Then:

$$EC50' = \left(\frac{\max(C)}{Eff'} - \max(C)\right) \Big/ \frac{clEff}{\max(clEff)} \qquad \text{equation [11]}$$

In this study, the set of drugs used for $EC50'$ calculation was the same set of drugs that were used for the intervention outcome optimization and are summarized in Table D in the S2 File.
Finally, the response values of each drug could be computed by using the corresponding $EC50'$, and rewriting equation 9 as shown in equation 12.

$$Eff_i' = \frac{C_i}{EC50' + C_i} \qquad \text{equation [12]}$$

As a result, a set of stimulus(P_i)-response(Eff_i') vector pairs could be computed for each of the drugs, one pair per each drug's protein target, and were added to the training set to construct the patient-specific QSP models.

Efficacy outcome – Clinical efficacy measure

ADHD was characterized and the tSginal of the subsequent protein set was chosen as model-derived efficacy measure. Then, the TPMS-based MoA models of the selected drug's clinical trials were built (summary in Table E in the S2 File), and the ADHD-tSignals were computed and used to measure optimization. In order to link the clinical efficacy measure, ADHD-RS IV, with the model-derived value, ADHD-tSignal, linear regression analysis between both variables was performed to parameterize the following equation:

$$Clinical\ efficacy\ measure = A * model - derived\ efficacy + B$$

equation [13]

The ADHD-tSignal strongly depends on the disease's molecular characterization. However, the initial bibliography-based definition could lead to the inclusion of proteins not related to the drugs under study or that might not have a clear role in the clinical manifestations affecting the clinical scale. Accordingly, the optimization process was centered by determining the molecular definition (ADHD protein subset) of the pathological condition whose tSignal would best correlate to clinically observed efficacies (ADHD-RS IV). This process was designed to maximize the absolute value of the Pearson correlation coefficient ($|\rho|$) between clinical and tSignal values, maintaining molecular information from the bibliography-based characterization. Thus, to identify the best A and B parameters in equation 13 that linked clinical efficacy measures with tSignals, proteins within the ADHD molecular definition were discarded iteratively until a molecular definition provided a strong correlation between both variables (8).

Supplementary figures

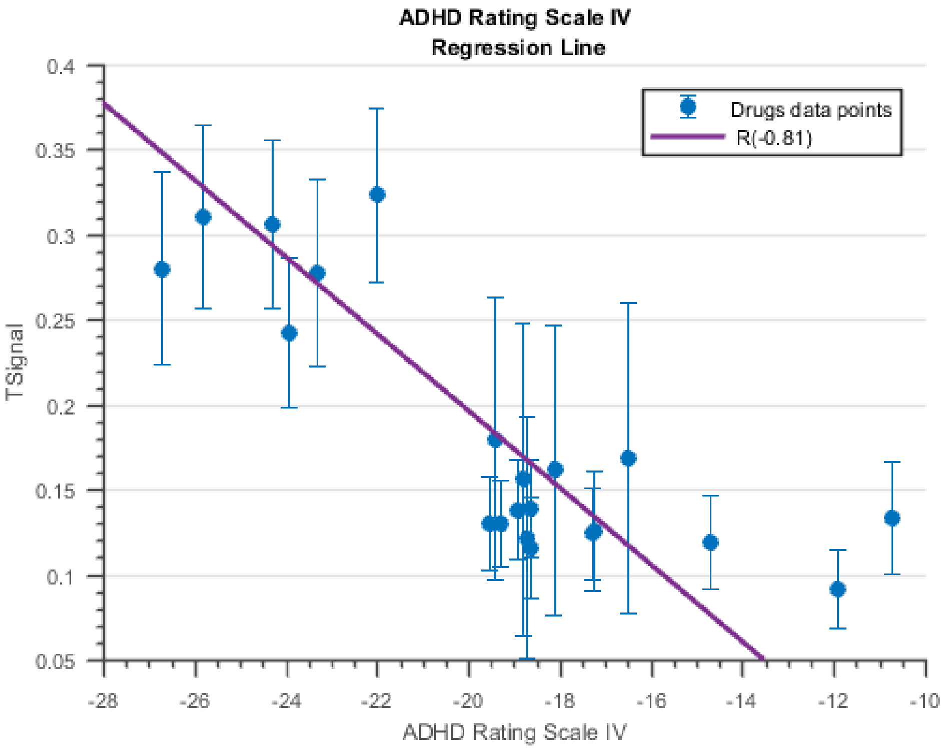

Figure A. Regression line between optimized ADHD tSignal in the pediatric-adolescent population in relation to ADHD-RS IV, change from baseline values (R = -0.81)

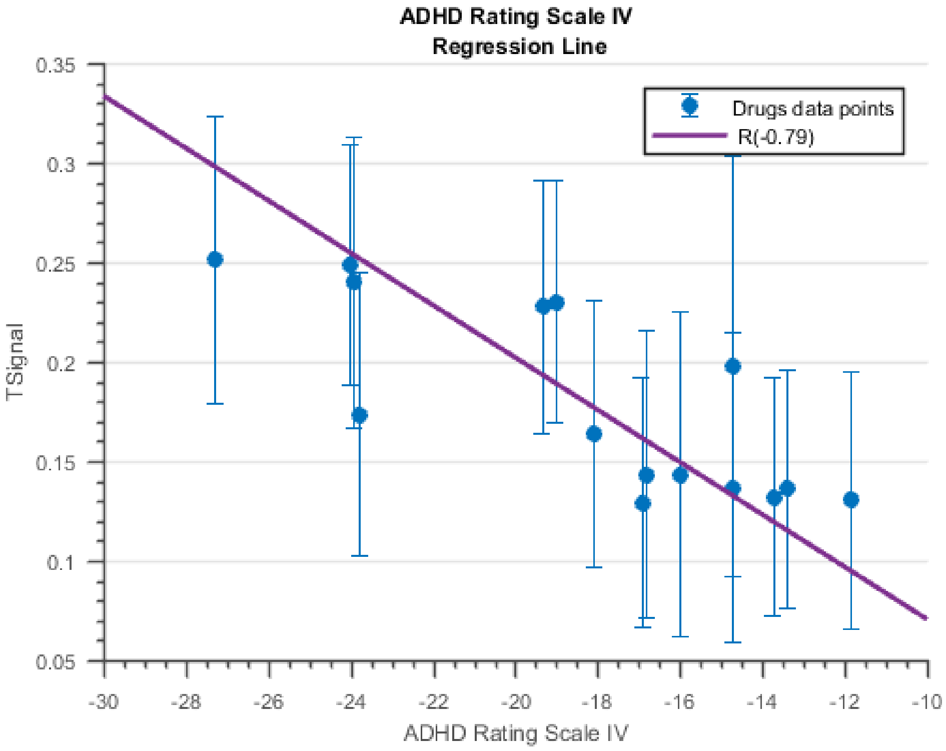

Figure B. Regression line between optimized ADHD tSignal in adult patients in relation to ADHD-RS IV, change from baseline values (R = -0.79)

References

1. Allen RJ, Rieger TR, Musante CJ. Efficient Generation and Selection of Virtual Populations in Quantitative Systems Pharmacology Models. *CPT Pharmacometrics Syst Pharmacol* (2016) **5**:140–146. doi:10.1002/psp4.12063

2. The European Social Survey. ESS Round 7: European Social Survey Round 7 Data (2014). Data file edition 2.2. NSD - Norwegian Centre for Research Data, Norway – Data Archive and distributor of ESS data for ESS ERIC. (2018) Available at: https://www.europeansocialsurvey.org/data/download.html?r=7

3. de Onis M, Onyango AW, Borghi E, Siyam A, Nishida C, Siekmann J. Development of a WHO growth reference for school-aged children and adolescents. *Bull World Health Organ* (2007) **85**:660–667. doi:10.2471/blt.07.043497

4. Halleyhit. Generate random numbers according to pdf or cdf. (2018) Available at: https://www.mathworks.com/matlabcentral/fileexchange/68492-generate-random-numbers-according-to-pdf-or-cdf

5. Chib S, Greenberg E. Understanding the Metropolis-Hastings Algorithm. *Am Stat* (1995) **49**:327–335. doi:10.1080/00031305.1995.10476177

6. Jorba G, Aguirre-Plans J, Junet V, Segu-Verges C, Ruiz JL, Pujol A, Fernandez-Fuentes N, Mas JM, Oliva B. In-silico simulated prototype-patients using TPMS technology to study a potential adverse effect of sacubitril and valsartan. *PLoS One* (2020) **15**:e0228926. doi:10.1371/journal.pone.0228926

7. Wagner JG. Kinetics of pharmacologic response. I. Proposed relationships between response and drug concentration in the intact animal and man. *J Theor Biol* (1968) **20**:173–201. doi:10.1016/0022-5193(68)90188-4

8. Akoglu H. User’s guide to correlation coefficients. *Turkish J Emerg Med* (2018) **18**:91–93. doi:10.1016/j.tjem.2018.08.001
